# Prefrontal-centered cortical alterations and network dysconnectivity in cocaine use disorder

**DOI:** 10.3389/fpsyt.2026.1897625

**Published:** 2026-08-21

**Authors:** Leonardo Melo Rothmann, Eduardo Tavares Portolan, Nathalia Bianchini Esper, Breno Sanvicente-Vieira, Augusto Martins Lucas Bittencourt, Thiago Wendt Viola, Pedro Eugênio Ferreira, Simon Fristed Eskildsen, Alexandre Rosa Franco, Rodrigo Grassi-Oliveira

**Affiliations:** 1 Translational Neuropsychiatry Unit, Department of Clinical Medicine, Graduate School of Health, Aarhus University, Aarhus, Denmark; 2 School of Medicine and Brain Institute of Rio Grande do Sul, Pontifical Catholic University of Rio Grande do Sul, Porto Alegre, Brazil; 3 Center for Strategic Data Initiatives, Child Mind Institute, New York, NY, United States; 4 Department of Psychology, Pontifical Catholic University of Rio de Janeiro, Rio de Janeiro, Brazil; 5 Center of Functionally Integrative Neuroscience (CFIN), Department of Clinical Medicine, Aarhus University, Aarhus, Denmark; 6 Center for Biomedical Imaging & Neuromodulation, Nathan S. Kline Institute for Psychiatric Research, Orangeburg, NY, United States; 7 Department of Psychiatry, Grossman School of Medicine, New York University, New York, NY, United States

**Keywords:** cocaine use disorder, multimodal magnetic resonance imaging, cortical thickness, cortical gyrification, prefrontal cortex, resting-state functional connectivity, brain connectome, network dysconnectivity

## Abstract

**Background:**

Cocaine use disorder (CUD) is a chronic, relapsing condition with substantial clinical burden, yet its neurobiological basis remains incompletely understood, particularly in the socially vulnerable populations most affected in real-world clinical settings. Existing neuroimaging evidence is fragmented across imaging modalities and derived largely from samples that underrepresent sustained adversity and polysubstance use. We used multimodal MRI to characterize structural and functional brain alterations in a clinically representative CUD sample.

**Methods:**

Seventy-two individuals with chronic cocaine use and 53 healthy controls underwent structural MRI and resting-state fMRI on a 3T scanner. Surface-based morphometry included cortical thickness, surface-based volume, and local gyrification index (a measure of the complexity of cortical folding), together with subfield-level volumetry of the hippocampus and amygdala. Functional connectivity was assessed using edge-wise general linear models. Analyses were adjusted for age, sex, and relevant covariates, with correction for multiple comparisons using cluster-wise and false discovery rate procedures.

**Results:**

CUD was associated with widespread cortical abnormalities, marked by reduced thickness and surface-based volume predominantly in prefrontal, temporal, and parietal cortices, with the strongest effects in the lateral orbitofrontal cortex, superior frontal gyrus, and inferior parietal lobule. Local gyrification index alterations were more focal and confined to frontal regions. No total hippocampal or amygdalar volume differences survived false discovery rate correction, although exploratory subfield analyses suggested localized increases in the hippocampal head, hippocampus-amygdala transition area, and corticoamygdaloid transition region. Functional connectome analysis identified four significant inter-network alterations: one hypoconnectivity between the left somatomotor network and dorsolateral prefrontal cortex and three hyperconnectivities between right somatomotor regions and medial frontal salience/ventral attention nodes.

**Conclusions:**

CUD is characterized by a predominantly prefrontal-centered cortical structural phenotype accompanied by selective frontal network dysconnectivity. The functional findings suggest a reweighting of large-scale networks favoring salience-driven action over prefrontal regulatory control, consistent with incentive-sensitization models of addiction. Observed in a treatment-seeking sample representative of real-world clinical populations, these results support frontal systems as a core locus of vulnerability in CUD and point to candidate targets for circuit-based intervention.

## Introduction

Cocaine use disorder (CUD) is a chronic, relapsing condition with no approved pharmacotherapy, making the brain systems that sustain compulsive use a central question for the field. Converging evidence from molecular, functional, and structural neuroimaging indicates that chronic substance exposure is associated with dysregulation of neurocircuits supporting reward valuation, salience attribution, executive control, and habit formation (1, 2). Neurobiological models of addiction, including incentive-sensitization (3) and impaired response inhibition (4) and salience attribution frameworks (5), emphasize that compulsive drug use emerges from an imbalance between sensitized motivational drive and weakened top-down control, instantiated across distributed cortical–subcortical networks.

Magnetic resonance imaging (MRI) studies in Substance use disorders (SUDs), including CUD, have consistently reported alterations in prefrontal, temporal, and parietal cortices, alongside disruptions in frontostriatal, salience, and sensorimotor networks (6–8). Structural MRI findings commonly include cortical thinning and reduced gyrification in regions implicated in executive control and decision-making, whereas resting-state functional MRI studies highlight abnormal coupling between control, salience, and habit-related systems (9–13). Together, these findings align with incentive-sensitization and impaired-response inhibition frameworks, which emphasize exaggerated motivational salience and deficient top-down regulation as core mechanisms of addiction (3, 14).

A critical gap, however, concerns the populations studied. Neuroimaging research in CUD has largely relied on convenience samples, often underrepresenting individuals exposed to sustained socioeconomic adversity (15), early-life stress, and polysubstance use (14, 16), as well sex differences (17, 18); factors known to shape brain development and stress-related neuroadaptations (19, 20). As a result, it remains unclear to what extent established MRI findings generalize to clinically and socially vulnerable populations, in whom addiction-related neurobiological alterations may be amplified, reorganized, or qualitatively distinct.

The present study aimed to expand and deepen our previous neuroimaging findings in CUD (21) by characterizing convergent alterations in cortical morphology and functional connectivity in male and female individuals using a multimodal imaging framework. We examined five complementary neuroimaging metrics. Four characterized brain structure: cortical thickness (the shortest distance between the gray-white matter boundary and the pial surface at each vertex, a sensitive index of the integrity of the cortical mantle), surface-based cortical volume (the product of cortical thickness and local surface area, jointly capturing the vertical [thinning] and tangential [areal] components of cortical morphology), the local gyrification index (the ratio of the buried, sulcal cortical surface to the outer, visible surface within a region, quantifying the degree of cortical folding, considering a feature largely established during early neurodevelopment), and hippocampal and amygdala subfield volumes (automatically segmented from the T1-weighted image; examined as an exploratory extension, given their small size and intrinsically lower signal-to-noise ratio). The fifth captured large-scale functional organization: resting-state functional connectivity (the temporal [Pearson] correlation of spontaneous blood-oxygen-level-dependent [BOLD] signal fluctuations between regions, indexing the functional coupling of distributed networks). Together with rigorous statistical control, these complementary metrics were used to characterize robust markers of cocaine-related brain alterations. By investigating convergent structural and functional alterations within the same individuals, and in a sample reflecting the social adversity and polysubstance use common in real-world CUD, this study seeks to delineate a more comprehensive neurobiological profile of the disorder and to identify frontal systems relevant to understanding vulnerability and informing future intervention.

## Methods

### Participants and MRI data acquisition

Participants with cocaine use disorder (CUD; n=72, 37 men and 35 women) were recruited during inpatient detoxification at public detoxification units. Eligibility required voluntary admission for detoxification, CUD as the primary diagnosis based on DSM-5, age ≥18 years, capacity to consent and complete assessments, no known neurological illness, and MRI suitability. Healthy controls (HC; n=53, 21 men and 32 women) were community volunteers who met MRI eligibility criteria, reported no lifetime substance use disorder (nicotine use disorder permitted), and had no neurological illness. Participants with CUD were scanned two weeks after admission to inpatient detoxification, under continuous supervised abstinence. Consistent with the cohort’s inclusion criteria, the sample comprised predominantly smoked cocaine users (~90% reported smoke cocaine use), most of whom also reported intranasal powder cocaine; intravenous use was essentially absent. Because smoke and powder use co-occurred in most participants, a formal route-stratified analysis was not feasible; detailed treatment procedures and cohort characterization are reported by Sanvicente‐Vieira, Rothmann (22). All participants provided written informed consent, and the study was approved by the Ethics Committee of the Pontifical Catholic University of Rio Grande do Sul in accordance with the Declaration of Helsinki.

MRI data were acquired on a 3T GE HDxt scanner with an eight-channel head coil. High-resolution T1-weighted anatomical images were acquired using the following parameters: repetition time (TR) = 6.1 ms, echo time (TE) = 2.18 ms, flip angle = 11°, slice thickness = 1 mm, number of excitations = 1, field of view = 256 mm, and in-plane resolution = 256 × 256. Resting-state functional MRI (rs-fMRI) data were acquired during an eyes-open session while participants were asked to fixate on a centralized white cross presented on a black screen. Functional images consisted of 210 volumes acquired using a gradient-echo EPI sequence (TR = 2000 ms, TE = 30 ms, flip angle = 90°, field of view = 240 mm, matrix = 64 × 64, 29 interleaved axial slices, slice thickness = 4.4 mm, in-plane resolution = 3.75 × 3.75 mm²). The first two volumes were discarded to allow for T1 signal stabilization, resulting in 208 usable volumes per participant.

### Structural MRI processing and surface-based morphometry

Anatomical images were processed using FreeSurfer version 7.4.1 (https://surfer.nmr.mgh.harvard.edu/), following the standard recon-all pipeline (23). Vertex-wise surface maps were generated for cortical thickness, the vertex-wise cortical ribbon element (volume), surface area, and the local gyrification index (LGI). Thickness was defined as the distance between the white and pial surfaces (mm). The ribbon element (also referred to as surface-based volume) was defined analytically as thickness × local surface area at each vertex (mm³). LGI was computed following Schaer, Cuadra (24) method and is unitless (area ratio) reflecting local sulcal complexity. Surface maps were resampled and smoothed with a 10-mm FWHM Gaussian kernel. In addition, total subcortical volumes were extracted for the hippocampus and amygdala, along with volumes of individual subfields based on the FreeSurfer segmentation protocols. Because the ribbon element depends on thickness × area, we report it as a secondary readout and center interpretation on thickness.

### Functional MRI preprocessing and connectivity analysis

Resting-state functional MRI data were preprocessed using the Configurable Pipeline for the Analysis of Connectomes (C-PAC, version 1.8.5) (25). The preprocessing steps included removal of the initial two volumes to allow for signal equilibration, followed by despiking using AFNI’s 3dDespike to attenuate large signal spikes. Head motion correction was then performed, and images were spatially normalized to the MNI152 standard space at 2 mm isotropic resolution. Functional brain masking was conducted using a combination of FSL and AFNI templates. Time series were band-pass filtered (0.01–0.10 Hz) to isolate low-frequency fluctuations relevant to resting-state connectivity.

Nuisance regression used two parallel strategies that shared the following steps: inclusion of head-motion parameters with temporal derivatives, quadratic terms, and their one-TR lagged versions, and inclusion of mean white-matter (WM) and cerebral spinal fluid (CSF) signals with the same derivative/quadratic/lagged expansions, plus the band-pass filter described above. The pipelines differed as follows: (i) a 36-parameter model that additionally included global signal regression [GSR] (global mean with derivatives, quadratic, and lagged terms); and (ii) an anatomical CompCor model that replaced GSR with five aCompCor principal components extracted from WM and CSF. Both strategies aimed to reduce physiological and motion-related confounds while preserving intrinsic connectivity (26). Following preprocessing, regional time series were extracted using the Schaefer parcellation (27), a cortical parcellation of 200 regions derived from resting-state functional connectivity and assigned to the 17 large-scale networks of Yeo-17 (28). For each participant, functional connectivity was quantified as the Pearson correlation between the mean time series of every pair of regions, yielding a 200 × 200 connectivity matrix whose 19,900 unique (off-diagonal) edges were entered into the edge-wise analyses.

### Quality control and participant exclusion

All participants were retained for the structural MRI morphometric analyses, except for the Local Gyrification Index (LGI) analysis, in which two females from the CUD and one male from the HC were excluded due to processing errors. For the resting-state functional MRI analyses (connectome), quality control procedures were applied to ensure robust signal and minimize motion-related artifacts. Participants were excluded if they did not complete the resting-state scan, if acquisition errors occurred, or if the data did not meet predefined quality thresholds. Specifically, only individuals with a mean framewise displacement (FD) ≤ 0.20 mm and a normalized cross-correlation ≥ 0.80 were included. These thresholds are based on current best practices in resting-state fMRI research and were applied consistently across groups. Consequently, six participants from the CUD group (1 male, 5 female) and two males from the HC group were excluded from the functional connectome analysis.

### Statistical analysis

Group comparisons were performed using vertex-wise general linear models separately for each morphometric modality. Statistical maps were performed using a vertex-wise cluster-forming threshold of p < 0.001 (two-tailed; 10-mm FWHM). Clusters were corrected for multiple comparisons using Monte Carlo simulation with a Cluster-wise p-value (CWP) of p < 0.05. For each significant cluster, participant-level mean values were obtained by averaging the corresponding morphometric map within the cluster mask on each participant’s smoothed map in the common template surface. Local gyrification index (LGI) models included group, age, and sex as predictors; cortical thickness and surface-based volume models additionally included estimated total intracranial volume (eTIV). For hippocampal and amygdalar analyses, total volumes were analyzed using ANCOVA models adjusting for age, sex, and *eTIV*. Subfield-level volumes were analyzed using separate ANCOVA models controlling for the total volume of the respective structure (hippocampus or amygdala), age, and sex. False discovery rate (Benjamini–Hochberg) correction was applied to p-values for the main group effect.

Group differences in functional connectivity were assessed using edge-wise general linear models across all 19,900 unique region-to-region connections. Each model included age, sex, and mean FD as covariates. The resulting beta coefficients and p-values were extracted for each edge. Multiple comparisons were controlled using the Benjamini–Hochberg false discovery rate (FDR) procedure, with statistical significance set at q < 0.05. Connections surviving this threshold were interpreted as significantly different between groups.

To address confounding by sociodemographic and polysubstance factors, we repeated the cluster- and edge-level comparisons adding, in turn, years of regular alcohol use, years of education, both jointly, CTQ total, and income, comparing baseline and adjusted models within identical complete-case samples. Socioeconomic status (SES) (education, income), sex, and early-life stress (ELS) (CTQ total) were further tested as group × moderator interactions for every significant cluster and edge (continuous moderators mean-centered; FDR within each family). For each participant, the mean morphometric value was extracted within each significant cortical cluster identified by the vertex-wise group comparisons (cortical thickness, surface-based volume, and LGI clusters). Each cluster value was z-scored across participants, and a single structural-alteration score was defined as the equally weighted mean of the z-scored cluster values. Because every cluster reflected a reduction in CUD, higher scores index a more preserved cortical profile. Modality-specific scores (thickness, volume, LGI) were derived identically over their respective clusters. The four significant connectome edges were each z-scored across participants and combined into a single functional-dysconnectivity score (FuncDys), sign-aligned so that higher values index a more CUD-like profile: FuncDys = [z(e2) + z(e3) + z(e4) − z(e1)]/4, where e1 is the somatomotor–dorsolateral-prefrontal hypoconnectivity (control > CUD; subtracted) and e2–e4 are the somatomotor–medial-frontal hyperconnectivities (CUD > control; added). Within-individual coupling was tested with a single ordinary-least-squares (OLS) model, FuncDys ~ structural-alteration score × group + age + sex + mean framewise displacement (sex and group as categorical factors), evaluating the structural-score main effect (individual-level coupling) and the structural-score × group interaction (group difference in coupling); within-group Pearson correlations are also reported. The model was estimated for the overall score and, secondarily, for each modality-specific score. A targeted cross-modal PLS related the structural block to the functional block within the significant results (clusters and edges). Both matrices were residualized for age, sex, and mean framewise displacement and z-scored feature-wise. We computed the cross-covariance matrix and its singular value decomposition, retaining the first latent variable (LV1); we report the proportion of cross-covariance explained by LV1, the correlation between the structural and functional LV1 scores, and the structural and functional loadings. Significance was assessed by permuting the rows of the functional block (2,000 permutations) and recomputing the leading singular value. Because both blocks were selected on the group contrast, this analysis is partially circular and is reported as a characterization of the joint pattern rather than as independent evidence of coupling. To provide a non-circular, out-of-sample test, a second PLS related the structural block to the whole-connectome node-degree profile (200 Schaefer-200 nodes; not selected on the group contrast), residualized and z-scored as above, using 5-fold cross-validation with significance assessed by permuting subject correspondence (500 permutations). We tested whether functional group effects concentrate near structurally altered cortex by comparing two maps: (a) the edge-wise group-effect magnitude across all 19,900 connectome edges, and (b) an edge proximity-to-altered-cortex map, defined per edge as the mean, across its two nodes, of each node’s proximity (the negative minimum Euclidean distance from the Schaefer-200 node centroid to the nearest significant structural-cluster peak (MNI space)). Association was quantified with Spearman’s rho and evaluated against a node-permutation null (2,000 permutations) and a spatially constrained spin null preserving spatial autocorrelation (1,000 random 3-D rotations of the node centroids with nearest-neighbor reassignment) (29). A complementary categorical test compared |t| for edges with ≥1 node within 15 mm of a structural cluster versus the remainder. Finally, an intensity-based signal-to-noise ratio (mean/SD of the normalized T1 within each label) was computed for each hippocampal/amygdalar subfield.

## Results

Sociodemographic and clinical data are presented in **Table 1**.

**Table 1:** Sociodemographic and clinical characteristics.

Sociodemographic	Cocaine use disorder group (n = 72)	Control group (n = 53)	P-value
Sex (female/male)	35/37	32/21	0.19
Age (years)	32.64 (± 5.65)	28.15 (± 6.33)	<0.001
Education	7.79 (± 2.64)	11.59 (± 1.84)	<0.001
Handedness (right-handed)	72	53	–
Race	–	–	–
Black	27 (42.9%)	12 (26.1%)	0.109
White	34 (54.0%)	34 (73.9%)	0.054
Mixed Race (Brown)	2 (3.2%)	0 (0.0%)	0.619
Annual Salary (U$D)	297.5 (± 802.7)	616.4 (± 801.5)	<0.001
Addiction severity index IV (ASI)			
Drugs	51.15 (± 5.43)	31.15 (± 0.77)	<0.001
Family and Child Support	54.12 (± 9.35)	53.79 (± 8.19)	0.998
Alcohol Use	49.35 (± 6.70)	43.77 (± 5.94)	<0.001
Psychiatric Status	47.65 (± 6.45)	38.00 (± 6.08)	<0.001
Medical Status	43.61 (± 7.76)	43.17 (± 7.02)	0.906
Legal Status	49.76 (± 5.63)	47.36 (± 3.53)	0.009
Employment	40.08 (± 3.90)	36.45 (± 4.47)	<0.001
Family and Social Support	68.04 (± 11.48)	50.04 (± 17.76)	<0.001
Substance Use Disorder (SUD)	–	–	–
SUD Treatments	4.26 (± 4.15)	–	–
Cocaine Age of First Use (years)	19.65 (± 5.86)	20.50 (± 6.36)	–
Cocaine Regular Use (years)	7.49 (± 6.62)	–	–
Cocaine Past Month Use (days)	14.76 (± 10.25)	-	
Alcohol Age of First Use (years)	14.94 (± 3.69)	16.83 (± 2.69)	<0.001
Alcohol Regular Use (years)	6.07 (± 7.40)	–	–
Alcohol Past Month Use (days)	6.76 (± 8.68)	2.15 (± 3.16)	0. 019
Any Illegal Drug Frequency (days/week)	5.60 (± 2.13)	–	–
Cannabis Age of First Use (years)	15.21 (± 3.57)	18.94 (± 3.70)	<0.001
Cannabis Regular Use (years)	7.71 (± 7.52)	–	–
Cannabis Past Month Use (days)	4.59 (± 8.68)	0.00	–
Tobacco Age of First Use (years)	13.52 (± 2.97)	17.15 (± 3.15)	<0.001
Tobacco Regular Use (years)	11.84 (± 9.90)	1.63 (± 4.22)	<0.001
Tobacco Past Month Use (days)	24.13 (± 10.43)	2.57 (± 8.52)	<0.001
Craving and Mood Symptoms			
Cocaine Selective Severity Assessment	25.03 (± 15.85)	–	–
Beck Depression Inventory-II (BDI-II)	11.26 (± 8.68)	5.47 (± 5.41)	0.001
Childhood Trauma Questionnaire (CTQ)			
CTQ Total	55.65 (± 15.28)	46.10 (± 10.44)	<0.001
Physical Neglect	8.44 (± 3.77)	6.10 (± 1.98)	<0.001
Emotional Neglect	11.52 (± 5.97)	8.31 (± 4. 12)	0.004
Sexual Abuse	7.23 (± 4.85)	5.79 (± 2.32)	0.207
Physical Abuse	9.21 (± 4.75)	7.10 (± 3.34)	0.004
Emotional Abuse	10.93 (± 5.21)	7.57 (± 3.44)	<0.001

Substance use values for the control group reflect non-pathological (experimental/occasional) lifetime use reported by a subset of controls; no control met criteria for any substance use disorder (nicotine use disorder permitted). Number of controls reporting any lifetime use: cocaine n = 2, cannabis n = 18, tobacco n = 32, alcohol n = 50; sustained regular use was rare (regular-use years > 0: cocaine 0, cannabis 0, tobacco 10, alcohol 0).

### Neuroanatomical differences

All the morphometric analyses (thickness, surface-based volume, and LGI) identified 50 significant clusters, symmetrically distributed across hemispheres (25 per side). Alterations were predominantly located in the frontal lobe (n = 27), with additional clusters in the parietal (n = 11), temporal (n = 8), and occipital (n = 4) cortices. Beyond group effects, several covariates showed significant associations. Age influenced cortical morphology in 19 clusters (8 thickness, 11 volume), consistent with normative age-related decline and age differences between groups. Sex effects emerged in seven clusters across thickness, volume, and LGI, with consistently higher values in males compared to females, in line with known sexual dimorphism. The cortical findings were robust to adjustment of the sociodemographic and polysubstance confounding factors, almost all results remained FDR-significant after adding SES, ELS, alcohol, and education.

A summary of main effects is presented in **Table 2**, while detailed cluster-level descriptions are provided in **Supplementary Table 1**. Sensitivity results are shown in **Supplementary Table 3**.

**Table 2:** Cluster-level morphometric differences between cocaine use disorder (CUD) and controls (HC).<i>.

Hemisphere	Region	HC	CUD	Statistics (df)
Local Gyrification Index				
Left	Lateral Orbitofrontal	2.88	2.77	F (1, 118) = 12.09, pFDR = 0.002, η² = 0.08
Right	Precentral	2.44	2.38	F (1, 118) = 5.26, pFDR = 0.035, η² = 0.04
Cortical Thickness				
Left	Inferior Temporal	2.75	2.58	F (1, 120) = 54.03, pFDR < 0.001, η² = 0.29
Left	Lateral Orbitofrontal	2.85	2.66	F (1, 120) = 53.13, pFDR < 0.001, η² = 0.28
Left	Caudal Middle Frontal	2.66	2.50	F (1, 120) = 36.30, pFDR < 0.001, η² = 0.22
Left	Rostral Middle Frontal	2.59	2.43	F (1, 120) = 36.89, pFDR < 0.001, η² = 0.22
Left	Rostral Middle Frontal	2.49	2.34	F (1, 120) = 32.52, pFDR < 0.001, η² = 0.20
Left	Supramarginal	2.44	2.29	F (1, 120) = 36.10, pFDR < 0.001, η² = 0.22
Left	Rostral Middle Frontal	2.48	2.35	F (1, 120) = 26.47, pFDR < 0.001, η² = 0.17
Left	Rostral Middle Frontal	2.77	2.58	F (1, 120) = 23.84, pFDR < 0.001, η² = 0.15
Left	Fusiform	2.50	2.36	F (1, 120) = 20.02, pFDR < 0.001, η² = 0.14
Left	Superior Parietal	2.46	2.31	F (1, 120) = 24.74, pFDR < 0.001, η² = 0.17
Left	Superior Frontal	3.12	2.94	F (1, 120) = 9.24, pFDR < 0.001, η² = 0.06
Left	Postcentral	2.85	2.66	F (1, 120) = 25.41, pFDR < 0.001, η² = 0.16
Left	Postcentral	2.52	2.30	F (1, 120) = 21.33, pFDR < 0.001, η² = 0.14
Left	Medial Orbitofrontal	2.57	2.39	F (1, 120) = 24.75, pFDR < 0.001, η² = 0.16
Left	Lateral Occipital	2.23	2.10	F (1, 120) = 10.52, pFDR = 0.001, η² = 0.07
Left	Lateral Occipital	2.46	2.30	F (1, 120) = 25.56, pFDR < 0.001, η² = 0.17
Left	Precentral	2.91	2.66	F (1, 120) = 12.10, pFDR < 0.001, η² = 0.09
Right	Middle Temporal	2.86	2.68	F (1, 120) = 49.29, pFDR < 0.001, η² = 0.27
Right	Inferior Parietal	2.59	2.42	F (1, 120) = 50.24, pFDR < 0.001, η² = 0.28
Right	Fusiform	2.34	2.15	F (1, 120) = 23.40, pFDR < 0.001, η² = 0.16
Right	Postcentral	2.34	2.20	F (1, 120) = 25.41, pFDR < 0.001, η² = 0.17
Right	Inferior Parietal	2.55	2.37	F (1, 120) = 38.75, pFDR < 0.001, η² = 0.23
Right	Lateral Orbitofrontal	2.69	2.53	F (1, 120) = 27.55, pFDR < 0.001, η² = 0.17
Right	Pars Opercularis	2.62	2.47	F (1, 120) = 21.35, pFDR < 0.001, η² = 0.14
Right	Lateral Occipital	2.38	2.18	F (1, 120) = 18.55, pFDR < 0.001, η² = 0.12
Right	Superior Parietal	2.52	2.27	F (1, 120) = 19.48, pFDR < 0.001, η² = 0.13
Right	Postcentral	2.80	2.62	F (1, 120) = 22.57, pFDR < 0.001, η² = 0.15
Right	Transverse Temporal	2.56	2.42	F (1, 120) = 13.54, pFDR < 0.001, η² = 0.09
Right	Supramarginal	2.47	2.32	F (1, 120) = 23.20, pFDR < 0.001, η² = 0.15
Right	Superior Temporal	2.30	2.15	F (1, 120) = 18.71, pFDR < 0.001, η² = 0.13
Right	Superior Frontal	2.80	2.57	F (1, 120) = 10.87, pFDR = 0.001, η² = 0.07
Right	Pars Triangularis	2.82	2.64	F (1, 120) = 19.25, pFDR < 0.001, η² = 0.13
Right	Lateral Orbitofrontal	2.99	2.82	F (1, 120) = 12.93, pFDR < 0.001, η² = 0.09
Right	Precentral	2.92	2.74	F (1, 120) = 14.05, pFDR < 0.001, η² = 0.10
Cortical Surface-Based Volume				
Left	Superior Frontal	2.85	2.53	F (1, 120) = 23.7521, pFDR < 0.001, η² = 0.11
Left	Lateral Orbitofrontal	2.34	2.01	F (1, 120) = 23.10, pFDR < 0.001, η² = 0.10
Left	Pars Triangularis	1.31	1.12	F (1, 120) = 15.29, pFDR < 0.001, η² = 0.09
Left	Lateral Orbitofrontal	3.57	3.18	F (1, 120) = 21.69, pFDR < 0.001, η² = 0.12
Left	Middle Temporal	3.20	2.62	F (1, 120) = 21.73, pFDR < 0.001, η² = 0.12
Left	Pars Opercularis	1.94	1.69	F (1, 120) = 12.59, pFDR < 0.001, η² = 0.08
Left	Superior Frontal	2.67	2.08	F (1, 120) = 13.11, pFDR < 0.001, η² = 0.09
Right	Lateral Orbitofrontal	2.19	1.92	F (1, 120) = 19.82, pFDR < 0.001, η² = 0.09
Right	Superior Frontal	3.27	2.87	F (1, 120) = 22.51, pFDR < 0.001, η² = 0.12
Right	Lingual	2.55	2.03	F (1, 120) = 21.29, pFDR < 0.001, η² = 0.14
Right	Middle Temporal	1.64	1.38	F (1, 120) = 17.68, pFDR < 0.001, η² = 0.09
Right	Postcentral	1.33	1.14	F (1, 120) = 13.36, pFDR < 0.001, η² = 0.08
Right	Superior Frontal	2.41	2.11	F (1, 120) = 11.44, pFDR < 0.001, η² = 0.05
Right	Rostral Middle Frontal	2.21	1.71	F (1, 120) = 16.92, pFDR < 0.001, η² = 0.11

Clusters were identified on vertex-wise maps using cluster-wise Monte Carlo correction (CWP < 0.001; cluster-forming > 3.0; 10-mm FWHM smoothing). The table reports unadjusted group means (HC, CUD) for each cluster. Statistics (df) gives the F-statistic for the main effect of group with its degrees of freedom, followed by the FDR-corrected p-value and partial eta-squared: F(1, df_2_); pFDR; η². All models adjust for age and sex; eTIV is additionally included for thickness and volume (not for LGI). Units: thickness (mm), surface-based volume (mm³), LGI (a.u.). Two-sided tests.

### Cortical thickness

**Figure 1:**
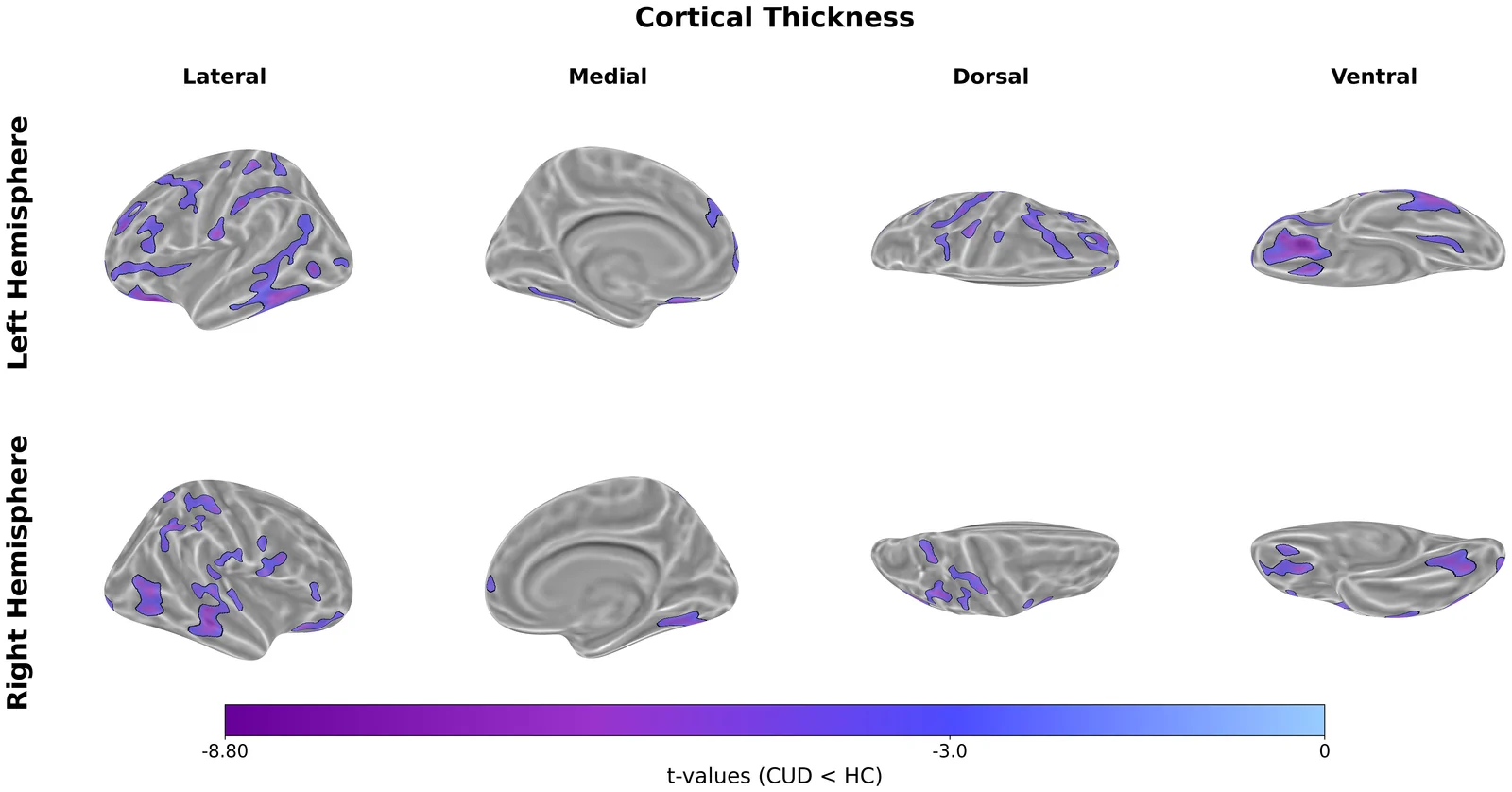
Cortical thickness results. Surface rendering of cortical regions showing cortical thinning in CUD compared to HC, displayed on the fsaverage template (super inflated surface) across lateral, medial, dorsal, and ventral views. Black contours denote cluster-wise significant areas (CWP < 0.001). Vertex-wise color maps use a dark purple–to–light blue gradient to represent t-values for CUD < HC, thresholded at |t| ≥ 3.0.

Widespread cortical thinning was observed across 34 clusters, encompassing prefrontal, parietal, temporal, and occipital cortices. Frontal effects were most prominent in inferior and middle frontal regions, whereas parietal alterations involved superior parietal and supramarginal cortices. Temporal reductions extended to fusiform and inferior temporal areas. These spatial patterns align with networks supporting executive control, attentional regulation, and emotional processing, functions frequently implicated in substance use disorders (**Figure 1**; **Table 2**; **Supplementary Table 1**).

### Volumetry

#### Cortical ribbon element

**Figure 2:**
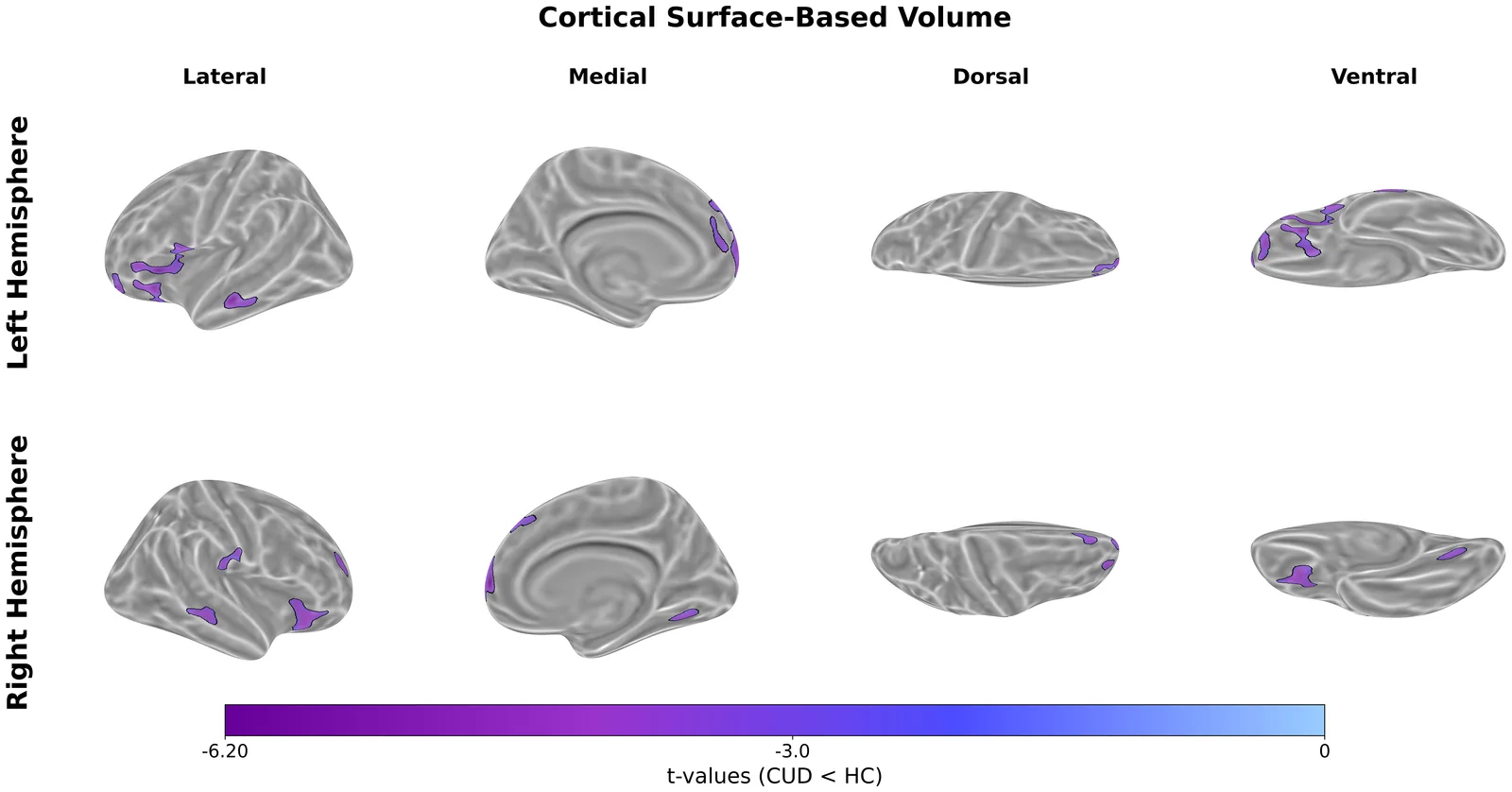
Cortical surface-based volume. Surface rendering of cortical regions showing reduced volume in CUD relative to HC, displayed on the fsaverage template (inflated surface) across lateral, medial, dorsal, and ventral views. Black contours outline regions surviving cluster-wise correction (CWP < 0.001), while the color maps display only vertices exceeding |t| ≥ 3.0. Vertex-wise color maps use a dark purple–to–light blue gradient to represent t-values for CUD < HC, thresholded at |t| ≥ 3.0.

Vertex-wise cortical ribbon element (surface-based volume; thickness × local surface area) showed 14 significant clusters, with a marked predominance in the frontal cortex, especially orbitofrontal and prefrontal regions. Additional disturbance were found in the temporal, parietal, and occipital cortices. These reductions overlapped extensively with thickness. Notably, surface area, analyzed with identical preprocessing and thresholds, yielded no FWE-significant clusters, indicating that ribbon-element effects largely recapitulate thickness rather than independent area changes. We therefore present the ribbon element as a secondary readout (**Figure 2**; **Table 2**; **Supplementary Table 1**).

### Subcortical structures

ANCOVA controlling for age, sex, and eTIV revealed no significant group differences in total hippocampal or amygdalar volumes after FDR correction. At the subfield level, uncorrected analyses suggested larger volumes in the CUD group bilaterally in the hippocampal head, including the molecular layer and hippocampal–amygdaloid transition area (HATA), as well as in the right corticoamygdaloid transition area of the amygdala. However, none of these findings survived FDR correction.

### Local gyrification index

**Figure 3:**
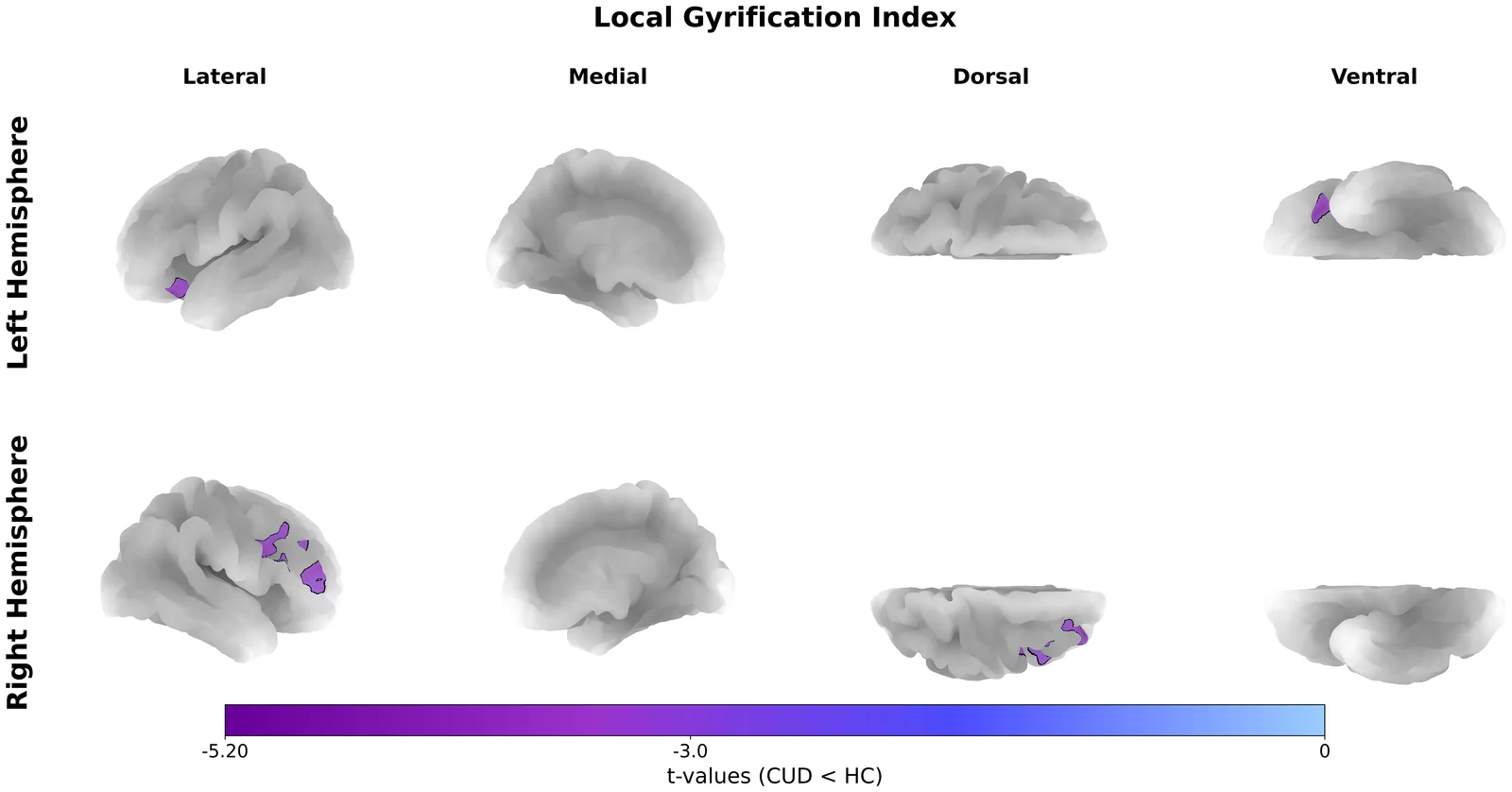
Local gyrification index (LGI). Surface rendering of cortical regions showing reduced local gyrification in individuals with cocaine use disorder (CUD) compared to healthy controls (HC), displayed on the fsaverage template (pial surface) across lateral, medial, dorsal, and ventral views. Black contours indicate regions surviving cluster-wise correction (CWP < 0.001). Vertex-wise color maps use a dark purple–to–light blue gradient to represent t-values for CUD < HC, thresholded at |t| ≥ 3.0.

Gyrification differences were restricted to two clusters in the frontal cortex. This focal pattern is consistent with the anatomical specificity of LGI, which reflects folding variations in circumscribed rather than widespread cortical regions. Despite their limited extent, these effects converge with thickness and volume alterations in adjacent prefrontal areas, further underscoring frontal vulnerability in cocaine use (**Figure 3**; **Table 2**; **Supplementary Table 1**).

### Overview of cortical morphological alterations

**Figure 4:**
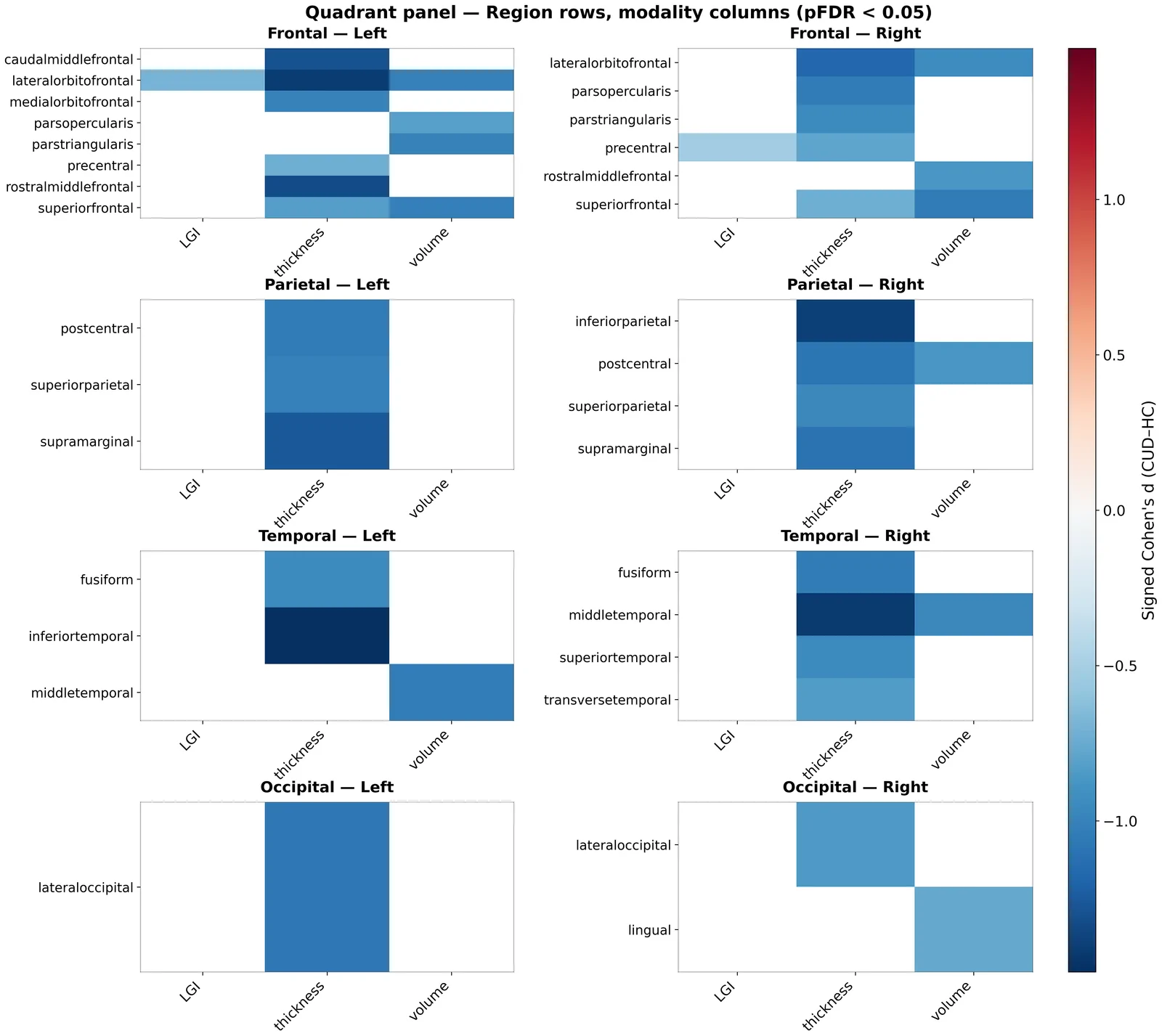
Quadrant panel summarizing all significant cortical morphological alterations (pFDR < 0.05). Heatmaps display effect sizes (signed Cohen’s d, CUD–HC) across lobes, hemispheres, and modalities (local gyrification index, cortical thickness, surface-based volume). Alterations were most widespread in frontal and temporal cortices, with convergent effects in bilateral orbitofrontal, precentral, fusiform, and lateral occipital regions. Thickness showed the greatest extent of reductions, while LGI effects were more circumscribed.

To integrate findings across morphometric modalities, we generated a quadrant panel summarizing all significant clusters (**Figure 4**). Predominant alterations were observed in frontal and temporal lobes, with more circumscribed involvement of parietal and occipital cortices. Bilateral effects were particularly evident in orbitofrontal, precentral, superior frontal, middle temporal, postcentral, fusiform, and lateral occipital regions. Among modalities, cortical thickness exhibited the most widespread reductions, whereas LGI alterations were sparse and localized. Notably, several regions showed cross-modal overlap between thickness and surface-based volume, underscoring pronounced structural vulnerability in CUD.

### Neurocircuitry differences

#### Connectome analysis

**Figure 5:**
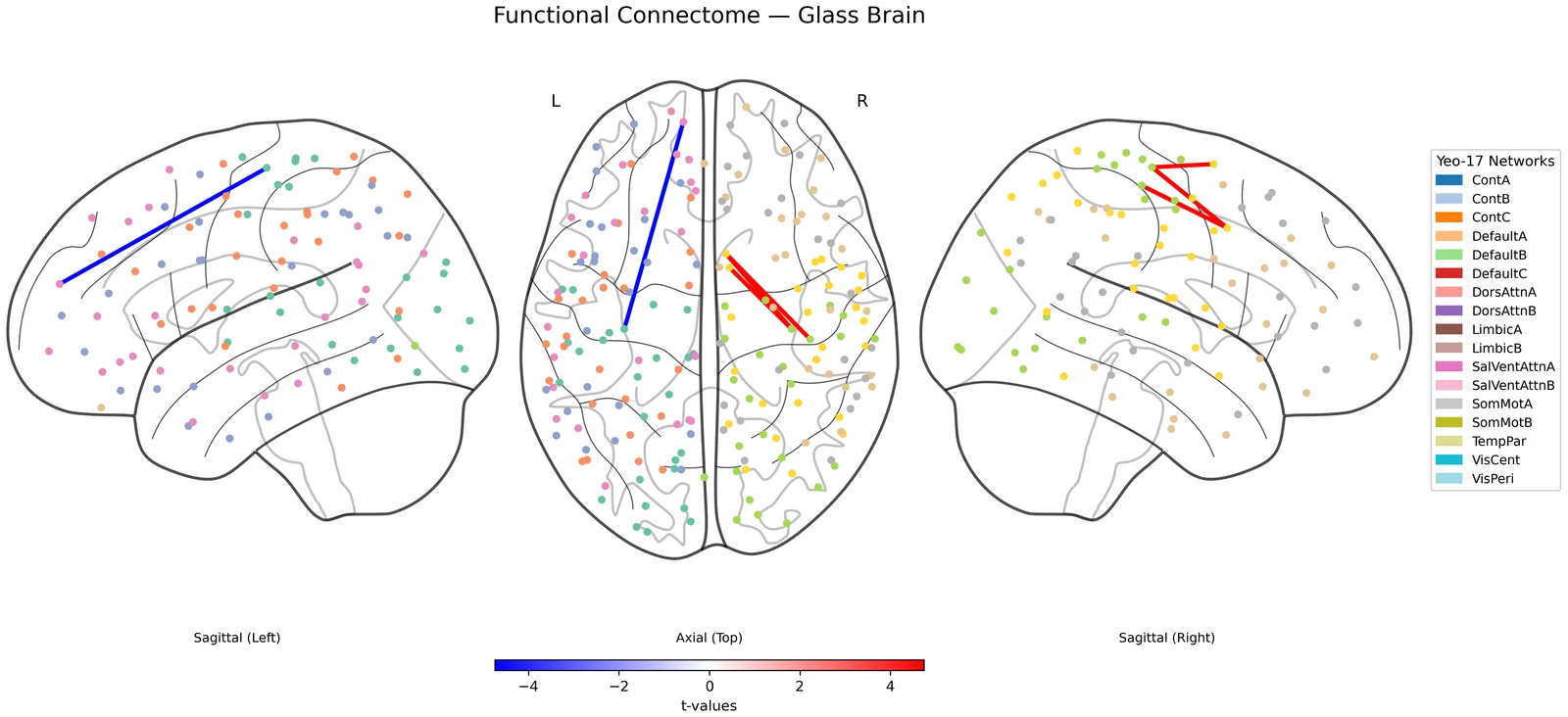
Functional connectome. Glass brain represents significant functional connectivity differences between the CUD and the HC groups. Nodes correspond to cortical regions from the Schaefer-200 parcellation, color-coded according to the 17-network Yeo functional atlas. Lines represent connections surviving false discovery rate correction (CWP < 0.05), with red indicating CUD > HC and blue indicating CUD < HC. Panels display sagittal (left and right) and axial (top) views. The color bar represents t-values for between-group differences.

Edge-wise analysis of resting-state functional connectivity revealed a selective pattern of inter-network disruptions in the CUD group relative to HC, after correction for multiple comparisons (FDR < 0.05). Four significant connections emerged as shown in **Figure 5**. One showed reduced connectivity between the left somatomotor network and the left dorsolateral prefrontal cortex, a node of the default mode network. This hypoconnectivity may indicate weakened coupling between motor outputs and prefrontal regulatory systems. In contrast, three connections exhibited increased functional connectivity in the CUD group, all involving right-hemisphere somatomotor regions and distinct nodes of the salience/ventral attention network, particularly within the right medial frontal cortex. This pattern points to atypical strengthening of communication between sensorimotor and salience-processing systems, consistent with the frontal vulnerability observed in structural analyses. The connectivity findings were robust to adjustment, all results remained FDR-significant after adding SES, ELS, alcohol, and education, as shown in **Supplementary Table 3**.

#### Sensitivity analysis

To verify that the prefrontal-centered findings were not driven by between-group differences in potential confounders, we re-estimated the group effect for every significant cluster and connectome edge after adding years of regular alcohol use, years of education, monthly income, and childhood trauma (CTQ total) as covariates (**Supplementary Table 3**). The cortical findings were robust: of the 50 clusters significant in the primary analysis, 50 remained FDR-significant after adjustment for income, 49 for alcohol use, 49 for childhood trauma, 47 for education, and 44 under the most conservative joint alcohol and education model; all four dysconnectivity edges remained significant under every adjustment. We further tested whether the group differences were moderated by sex, socioeconomic status (education and income), or early-life stress (CTQ total) by adding group × moderator interaction terms to each model (**Supplementary Table 4**). No interaction survived FDR correction for any moderator in either modality.

### Multimodal convergence of structural and functional alterations

When considered together, structural and functional results revealed a convergent pattern of brain alterations in cocaine use disorder. Morphological findings indicated widespread cortical thinning, particularly in frontal and temporal regions, with additional circumscribed effects in parietal and occipital cortices. The functional findings were more selective, comprising a circumscribed set of somatomotor–frontal disconnections involving dorsolateral-prefrontal and medial-frontal salience nodes. Although both phenotypes center on frontal systems, their correspondence proved weak when tested directly, indicating that the structural and functional alterations carry complementary rather than redundant information.

In the complete-modality subsample (N = 114; 64 CUD, 50 HC), the structural-alteration score did not predict functional dysconnectivity (β = 0.02, p = 0.96), and this relationship did not differ between groups (p = 0.90); within-group associations were negligible (CUD r = 0.05, p = 0.68; HC r = 0.03, p = 0.83). Modality-specific scores (thickness, volume, LGI) yielded the same null pattern (all p > 0.4; **Supplementary Table 6**). A cross-validated PLS relating the structural clusters to the whole-connectome node-degree profile was also non-significant (p = 0.27). The first latent variable of the targeted structure-function PLS accounted for 92.1% of the cross-covariance between the two blocks, with a latent correlation of r = 0.42 between the structural and functional scores (p < 0.001). The functional loadings spanned all four edges (e3 = 0.58, e4 = 0.52, e2 = 0.46, e1 = −0.42) and the structural loadings were broadly distributed across thickness and volume clusters (**Supplementary Table 7**). Because both blocks were selected on the group contrast, this latent dimension re-expresses the shared group difference and is therefore interpreted as a descriptive characterization of the joint pattern rather than as independent evidence of individual-level coupling. Across all 19,900 edges, the magnitude of the functional group effect showed only a weak spatial association with proximity to structurally altered cortex (rho = 0.05). This association was significant under a node-permutation null (p = 0.007) but did not survive a spatially constrained spin null that preserves spatial autocorrelation (p = 0.09; **Supplementary Figure 1**); edges with ≥1 node within 15 mm of a structural cluster showed marginally higher group-effect magnitude than the remainder (p = 0.002). Because non-spin spatial permutations are inflated by autocorrelation, we interpret this spatial correspondence as suggestive at most. Together, these analyses indicate that the structural and functional phenotypes of CUD are largely complementary: they co-localize only weakly at the systems level and show no reproducible coupling at the individual level, suggesting the two modalities capture partially independent facets of the disorder rather than redundant expressions of a single process.

## Discussion

This multimodal neuroimaging study identified a convergent pattern of brain alterations in cocaine use disorder (CUD), characterized by widespread cortical structural abnormalities and a more selective profile of functional dysconnectivity. The most robust signal was a bilateral cortical phenotype centered on prefrontal regions and extending into temporal and parietal association cortices, expressed primarily as cortical thinning and, to a lesser extent, reduced surface-based volume and focal hypogyrification. Resting-state analyses revealed a circumscribed set of connectivity abnormalities linking somatomotor regions with frontal nodes of the default mode and salience/ventral attention systems. In contrast, subcortical volumetric findings were limited and did not survive correction for multiple comparisons. Taken together, these results support a systems-level account of CUD in which the most reproducible abnormalities are cortical and network-based, particularly in circuits involved in executive regulation, salience processing, and action control.

### Cortical phenotype in cocaine use disorder

The structural results point most clearly to a prefrontal-centered cortical phenotype. Cortical thinning was the most extensive and consistent morphometric abnormality, with prominent involvement of the lateral orbitofrontal cortex, superior and middle frontal gyri, inferior parietal lobule, supramarginal cortex, and temporal association regions. Surface-based volume reductions showed substantial spatial overlap with thickness effects, whereas local gyrification abnormalities were sparse and confined to frontal cortex. This pattern indicates that the cortical signal in this sample is driven primarily by alterations in cortical thickness rather than by broad surface area changes or diffuse abnormalities in cortical folding (30).

The anatomical distribution of these findings is highly consistent with current neurobiological models of addiction. The lateral orbitofrontal cortex is critically involved in reward valuation, reversal learning, and the updating of behavior when contingencies shift, processes that are repeatedly disrupted in substance use disorders and are central to maladaptive persistence despite adverse outcomes (4, 31). Superior and middle frontal territories, including dorsolateral and dorsomedial prefrontal regions, support cognitive control, action selection, performance monitoring, and top-down regulation of behavior, and have been consistently implicated in impaired in inhibitory control and reduced self-regulation in cocaine addiction (1, 4, 31). Inferior parietal and supramarginal cortices belong to frontoparietal systems that allocate attention, integrate goal-relevant information, and support adaptive reorienting when internal states or environmental demands change; abnormalities in these regions fit well with the attentional bias, impaired flexibility, and cue-driven responding that characterize CUD (31, 32). Temporal association cortices contribute to higher-order integration of perceptual, emotional, and contextual information, and have also been linked to learned salience and drug-cue processing in cocaine dependence. Taken together, this anatomical pattern suggests that CUD is associated with abnormalities in distributed cortical systems required for adaptive self-regulation, rather than with dysfunction confined to a single executive or reward-related region. However, the current findings are not most parsimoniously interpreted as evidence of a generalized prefrontal deficit. Instead, they point to abnormalities in a more specific set of frontal regions implicated in behavioral regulation under conditions of motivational conflict and uncertainty. This interpretation fits with contemporary accounts of addiction emphasizing that executive dysfunction is multidimensional and embedded within broader frontoparietal systems.

### Morphometric specificity: thickness, volume, and gyrification

The relative dissociation across morphometric measures is itself informative. The predominance of cortical thickness effects, together with the absence of significant surface area differences, suggests that the structural phenotype in CUD is expressed primarily through alterations in cortical thickness rather than through broad areal reconfiguration. This interpretation is consistent with prior work in cocaine users and supports the view that the surface-based volume signal largely recapitulates thickness-related effects (33–35). By contrast, the focal nature of the gyrification findings suggests a partially distinct process. Because gyrification is shaped predominantly during prenatal and early postnatal development and is comparatively stable later in life, while cortical thickness remains responsive to later synaptic and dendritic remodeling (36–38), the coexistence of focal hypogyrification and widespread thinning is compatible with a model in which developmental liability and chronic cocaine-related neuroadaptation both contribute to the observed cortical phenotype. This developmental interpretation is especially relevant because many of the affected regions, particularly prefrontal and inferior parietal association cortices, mature relatively late (39). These regions undergo prolonged refinement of synaptic organization, myelination, and network specialization through adolescence and early adulthood, making them plausible sites both of premorbid neurodevelopmental variation and of substance-related remodeling during periods of heightened plasticity (39). Longitudinal developmental studies will be necessary to determine whether these features are antecedents, consequences, or both.

### Functional network alterations and salience-to-action coupling

The resting-state findings complement the structural abnormalities but are notably more selective. Instead of a diffuse pattern of connectome-wide disruption, CUD was associated with a circumscribed set of inter-network alterations linking somatomotor regions with frontal control and salience systems. The observed reduction in somatomotor-dorsolateral prefrontal coupling is consistent with diminished integration between action-related systems and regions supporting executive regulation, whereas increased coupling between somatomotor and medial frontal salience/ventral attention nodes suggests enhanced interaction between action systems and circuitry involved in assigning behavioral relevance (32, 40, 41). This profile supports the interpretation that functional abnormalities in CUD may reflect altered coordination between executive control and salience-driven action processes rather than nonspecific network disorganization. This functional profile is also compatible with addiction models centered on incentive sensitization (3). These models propose that compulsive drug seeking reflects not only deficient control, but also amplification of the motivational significance of drug-related cues and internal states. While resting-state fMRI cannot directly assess cue-triggered wanting (40), the present findings are consistent with a network configuration in which salience-related processes are more tightly linked to action systems.

### Stress, uncertainty, and contextual modulation

The functional profile observed here may also be considered in relation to broader models of chronic stress and environmental instability in addiction (15, 42). Prolonged adversity has been linked to altered large-scale organization of salience and control systems, often favoring stronger bottom-up processing and weaker top-down regulation (43). Under such conditions, environmental uncertainty may enhance the impact of salient cues and increase the tendency for motivationally relevant information to capture attention and influence behavior (15, 44). These processes are particularly relevant in populations exposed to socioeconomic instability, chronic threat, or unpredictable reward environments (45).

Critically, however, our data bound this contextual account. Although socioeconomic status, sex, and early-life stress motivated the study and were directly assessed, the prefrontal-centered phenotype proved robust to and independent of them: group differences survived adjustment for education, income, alcohol use, and childhood trauma, and were not moderated by sex, SES, or early-life stress (**Supplementary Tables 3, 4**). Rather than indicating that adversity shaped the findings, this suggests that the frontal structural–functional signature is a core, cross-cutting feature of CUD rather than a by-product of socioeconomic disadvantage or trauma load. The broader stress-and-uncertainty framework therefore describes a context in which this phenotype is expressed and with which it may interact, not a moderating influence we were able to detect, a distinction that larger, adequately powered samples should test directly.

### Strengths, limitations, and future directions

A key strength of this study is the integration of structural and functional neuroimaging within the same sample, which allowed convergent abnormalities to be examined across complementary levels of brain organization. The correspondence between prefrontal-centered cortical alterations and selective network-level dysconnectivity increases confidence that frontal systems are centrally involved in CUD, rather than emerging as modality-specific or isolated findings.

Several limitations should be considered. First, and most importantly, the cross-sectional design cannot establish temporal sequence or causality: the observed abnormalities may reflect antecedent vulnerability, neuroadaptations related to chronic cocaine exposure, or their cumulative interaction over time. Preclinical models help bridge this gap, as they permit longitudinal designs with pre-exposure baselines and controlled drug histories. In rats, resting-state connectivity acquired before and after cocaine self-administration reveals progressive cortico-striatal–limbic neuroadaptations absent at baseline, indicating that connectivity changes can be a consequence of cocaine exposure (46). Convergently, cocaine self-administration reduces prefrontal–accumbens connectivity, leading the authors to note that cross-sectional human findings may reflect the consequence rather than an antecedent of dependence (47). In nonhuman primates, chronic cocaine self-administration produces residual functional deficits concentrated in prefrontal, parietal, and cingulate cortex that persist into abstinence (48), and structural MRI in rodents links cocaine taking to gray-matter variation in medial prefrontal cortex (49). Together, these controlled-exposure models indicate that chronic cocaine can causally induce frontal structural and connectivity changes anatomically convergent with the phenotype observed here, supporting a contribution of cocaine exposure beyond pre-existing vulnerability alone. Critically, resolving this question in humans will require prospective longitudinal designs within pediatric cohorts assessed before and after the onset of substance use and the emergence of SUD. Because the affected prefrontal and association cortices continue to mature into early adulthood, such designs are uniquely positioned to disentangle premorbid neurodevelopmental liability from substance-related remodeling and to establish the timing of brain changes relative to use onset. Second, polysubstance use and clinical heterogeneity reduce the ability to attribute the findings specifically to cocaine, although this feature also increases ecological validity and reflects the reality of treatment-seeking and community CUD populations. Third, the functional findings were relatively circumscribed, which supports specificity but argues against broader claims of diffuse connectome-wide disruption. Fourth, the subcortical findings should be interpreted as exploratory: neither total hippocampal nor amygdalar volume differed after correction, and subfield-level signals did not survive FDR correction. This caution is reinforced by a signal-to-noise analysis: these small subfields are inherently low-SNR structures, and SNR was significantly lower in the CUD group than in controls across most subfields (**Supplementary Table 5**). Finally, the absence of direct behavioral and neurochemical measures limits mechanistic inference. In particular, the present design cannot determine whether the observed network alterations relate more closely to impaired inhibitory control, altered salience attribution, dopaminergic dysfunction, neuroinflammatory processes, or their interaction.

### Conclusion

In summary, this study indicates that cocaine use disorder is characterized by a convergent multimodal profile marked by widespread prefrontal-centered cortical abnormalities and selective alterations in salience-somatomotor connectivity. The strongest evidence supports a predominantly cortical phenotype involving systems necessary for executive regulation, attentional control, and action selection. Functional findings further suggest that CUD is associated with a reweighting of large-scale networks in which salience-related influences may gain greater access to action systems while prefrontal regulatory integration is weakened. This profile is consistent with an addiction framework in which persistent drug use and relapse vulnerability emerge from the interaction between frontoparietal structural vulnerability and heightened salience-driven action readiness.

## Data Availability

The datasets presented in this article are not readily available due to institutional, ethical, and legal restrictions in Brazil. Requests to access the datasets should be directed to rodrigo.grassi@pucrs.br.
