# Supplementary material for: Prefrontal-centered cortical alterations and network dysconnectivity in cocaine use disorder
Source: Front Psychiatry. 2026 Aug 21;17:1897625. doi: 10.3389/fpsyt.2026.1897625 (PMC13542897; doi:10.3389/fpsyt.2026.1897625)
Supplement: Supplementary file 1 [file Table1.docx]

**Supplementary Information**

Table S1. Morphological Results – detailed description

| Hemisphere | | Region | MNI_x | MNI_y | MNI_z | Cluster Area (mm²) | Number of Vertices | Peak t-value | CWP | HC | CUD | AdjDiff  (CUD-HC) | Statistics (df) | Eta^2^ | p_age | p_gender | p_eTIV |
| --- | --- | --- | --- | --- | --- | --- | --- | --- | --- | --- | --- | --- | --- | --- | --- | --- | --- |
| **LGI** | |  |  |  |  |  |  |  |  |  |  |  |  |  |  |  |  |
|  | Left | lateral orbitofrontal | -31.8 | 27.9 | -17.2 | 376.21 | 762 | -4.51 | 0.047 | 2.8811 | 2.7726 | 0.1063 | F (1, 118) = 12.0998, pFDR = 0.002, η² = 0.08 | 0.0888 | 0.274118 | 0.028126 |  |
|  | Right | precentral | 38.2 | 2.7 | 28.6 | 1046.3 | 2106 | -4.56 | 0.0002 | 2.4415 | 2.3841 | 0.0497 | F (1, 118) = 5.2692, pFDR = 0.0352087, η² = 0.0423 | 0.0423 | 0.260227 | 0.816621 |  |
|  | Right | rostralmiddlefrontal | 33.8 | 48.2 | 12.3 | 1005.15 | 1601 | -5.15 | 0.0002 | 2.2683 | 2.2312 | 0.0277 | F (1, 118) = 1.7532, pFDR = 0.18803011, η² = 0.0144 | 0.0144 | 0.142359 | 0.682869 |  |
| **Cortical Thickness** | |  |  |  |  |  |  |  |  |  |  |  |  |  |  |  |  |
|  | Left | inferiortemporal | -56.2 | -51.4 | -16.9 | 2328.46 | 4248 | -6.68 | 0.0001 | 2.754 | 2.5854 | 0.1507 | F (1, 120) = 54.0322, pFDR = 0.00000000061341561, η² = 0.292 | 0.292 | 0.008945 | 0.140845 | 0.190503 |
|  | Left | lateralorbitofrontal | -26.3 | 24.2 | -17 | 1227.29 | 2253 | -8.795 | 0.0001 | 2.8534 | 2.6694 | 0.1799 | F (1, 120) = 53.1397, pFDR = 0.00000000061341561, η² = 0.286 | 0.286 | 0.06903 | 0.003293 | 0.574973 |
|  | Left | caudalmiddlefrontal | -39.2 | 1.9 | 49.6 | 987.04 | 1663 | -4.871 | 0.0001 | 2.6675 | 2.5037 | 0.1508 | F (1, 120) = 36.3028, pFDR = 0.000000087667486, η² = 0.2289 | 0.2289 | 0.19293 | 0.889994 | 0.461636 |
|  | Left | rostralmiddlefrontal | -37.3 | 42.8 | -1.7 | 781.5 | 1468 | -4.902 | 0.0001 | 2.5954 | 2.4393 | 0.1387 | F (1, 120) = 36.8941, pFDR = 0.000000085637923, η² = 0.2257 | 0.2257 | 0.020095 | 0.385694 | 0.590128 |
|  | Left | rostralmiddlefrontal | -26.7 | 38 | 18.9 | 702.33 | 1151 | -6.667 | 0.0001 | 2.4935 | 2.3459 | 0.1242 | F (1, 120) = 32.524, pFDR = 0.00000032550354, η² = 0.2006 | 0.2006 | 0.015564 | 0.985958 | 0.060182 |
|  | Left | supramarginal | -51.7 | -25.8 | 31.5 | 644.68 | 1788 | -6.375 | 0.0001 | 2.4432 | 2.2942 | 0.1408 | F (1, 120) = 36.103, pFDR = 0.000000087667486, η² = 0.2262 | 0.2262 | 0.964906 | 0.182083 | 0.193135 |
|  | Left | rostralmiddlefrontal | -40.5 | 27.1 | 13.6 | 497.51 | 847 | -4.71 | 0.0001 | 2.4895 | 2.3523 | 0.1236 | F (1, 120) = 26.4778, pFDR = 0.0000032558835, η² = 0.1773 | 0.1773 | 0.181454 | 0.892271 | 0.313445 |
|  | Left | rostralmiddlefrontal | -18.9 | 61.2 | -2.9 | 421.91 | 629 | -4.684 | 0.0001 | 2.7748 | 2.5876 | 0.166 | F (1, 120) = 23.8491, pFDR = 0.0000064977798, η² = 0.1531 | 0.1531 | 0.005238 | 0.110337 | 0.259064 |
|  | Left | fusiform | -30.7 | -54.7 | -12.1 | 272.46 | 459 | -4.237 | 0.0019 | 2.5013 | 2.3646 | 0.1296 | F (1, 120) = 20.0256, pFDR = 0.000025864818, η² = 0.142 | 0.142 | 0.838588 | 0.531991 | 0.448751 |
|  | Left | superiorparietal | -34.1 | -50.3 | 61.7 | 259.15 | 554 | -4.543 | 0.0024 | 2.4611 | 2.312 | 0.1477 | F (1, 120) = 24.7403, pFDR = 0.0000046996017, η² = 0.1707 | 0.1707 | 0.77832 | 0.939997 | 0.737856 |
|  | Left | superiorfrontal | -6.6 | 47.8 | 37.7 | 240.31 | 395 | -4.365 | 0.0032 | 3.1226 | 2.9403 | 0.1269 | F (1, 120) = 9.2427, pFDR = 0.002903335, η² = 0.0632 | 0.0632 | 0.000154 | 0.358441 | 0.378531 |
|  | Left | postcentral | -62.6 | -15.1 | 16.5 | 214.7 | 562 | -6.586 | 0.0059 | 2.8564 | 2.6651 | 0.1794 | F (1, 120) = 25.4107, pFDR = 0.0000040293424, η² = 0.1671 | 0.1671 | 0.112935 | 0.04815 | 0.692054 |
|  | Left | postcentral | -35.7 | -33.1 | 65.5 | 213.95 | 570 | -6.300 | 0.0059 | 2.52 | 2.3083 | 0.1889 | F (1, 120) = 21.3383, pFDR = 0.000015072572, η² = 0.1467 | 0.1467 | 0.52979 | 0.135407 | 0.234876 |
|  | Left | medialorbitofrontal | -9 | 24.6 | -19.5 | 181.71 | 417 | -6.693 | 0.0157 | 2.577 | 2.3985 | 0.1734 | F (1, 120) = 24.7589, pFDR = 0.0000046996017, η² = 0.1666 | 0.1666 | 0.423484 | 0.104339 | 0.486694 |
|  | Left | lateraloccipital | -39.8 | -82.4 | 4.2 | 169.3 | 269 | -3.631 | 0.0218 | 2.2381 | 2.102 | 0.1169 | F (1, 120) = 10.5287, pFDR = 0.0015691844, η² = 0.0789 | 0.0789 | 0.262196 | 0.961256 | 0.212273 |
|  | Left | lateraloccipital | -41.6 | -63.9 | 0.3 | 161.03 | 296 | -6.542 | 0.0266 | 2.4624 | 2.3049 | 0.1498 | F (1, 120) = 25.5633, pFDR = 0.0000040293424, η² = 0.1749 | 0.1749 | 0.52719 | 0.962455 | 0.686215 |
|  | Left | precentral | -34.5 | -18.1 | 66.5 | 141.96 | 325 | -4.119 | 0.042 | 2.9183 | 2.6682 | 0.2383 | F (1, 120) = 12.1044, pFDR = 0.00076967632, η² = 0.0904 | 0.0904 | 0.348387 | 0.36165 | 0.798739 |
|  | Right | middletemporal | 64.3 | -27.1 | -11.4 | 1175.33 | 2399 | -8.195 | 0.0001 | 2.8648 | 2.6872 | 0.1718 | F (1, 120) = 49.2906, pFDR = 0.0000000012117755, η² = 0.2793 | 0.2793 | 0.093541 | 0.051649 | 0.487374 |
|  | Right | inferiorparietal | 44.8 | -62.5 | 5.4 | 841.39 | 1388 | -6.185 | 0.0001 | 2.5972 | 2.4207 | 0.1726 | F (1, 120) = 50.2436, pFDR = 0.0000000011463103, η² = 0.2824 | 0.2824 | 0.372468 | 0.023697 | 0.20954 |
|  | Right | fusiform | 29.5 | -73.7 | -9.3 | 792.66 | 1209 | -6.454 | 0.0001 | 2.342 | 2.1527 | 0.1752 | F (1, 120) = 23.406, pFDR = 0.0000074378076, η² = 0.1619 | 0.1619 | 0.430605 | 0.486664 | 0.809459 |
|  | Right | postcentral | 47.8 | -24.9 | 57 | 613 | 1494 | -6.000 | 0.0001 | 2.3494 | 2.2011 | 0.1379 | F (1, 120) = 25.4126, pFDR = 0.0000040293424, η² = 0.1733 | 0.1733 | 0.521882 | 0.584751 | 0.491583 |
|  | Right | inferiorparietal | 45.7 | -53.3 | 43.4 | 544.14 | 1269 | -5.907 | 0.0001 | 2.5569 | 2.3729 | 0.1623 | F (1, 120) = 38.7532, pFDR = 0.000000049805302, η² = 0.2369 | 0.2369 | 0.085748 | 0.464375 | 0.25881 |
|  | Right | lateralorbitofrontal | 31.6 | 26.7 | -18.8 | 534.06 | 885 | -6.492 | 0.0001 | 2.6998 | 2.5391 | 0.1374 | F (1, 120) = 27.5522, pFDR = 0.0000022756761, η² = 0.1709 | 0.1709 | 0.001068 | 0.152126 | 0.558176 |
|  | Right | parsopercularis | 40 | 8.2 | 21.5 | 409.73 | 908 | -5.507 | 0.0001 | 2.6224 | 2.4738 | 0.1294 | F (1, 120) = 21.3511, pFDR = 0.000015072572, η² = 0.1464 | 0.1464 | 0.147092 | 0.997065 | 0.127926 |
|  | Right | lateraloccipital | 24.7 | -97.6 | -12.9 | 292.45 | 350 | -5.432 | 0.0002 | 2.3877 | 2.1898 | 0.1979 | F (1, 120) = 18.5511, pFDR = 0.000042840606, η² = 0.1296 | 0.1296 | 0.391421 | 0.055801 | 0.730751 |
|  | Right | superiorparietal | 26 | -55.6 | 63.6 | 286.74 | 651 | -5.809 | 0.0002 | 2.5292 | 2.2778 | 0.2301 | F (1, 120) = 19.4893, pFDR = 0.00003153627, η² = 0.1378 | 0.1378 | 0.181805 | 0.989666 | 0.6823 |
|  | Right | postcentral | 62.6 | -11.3 | 25.2 | 275.76 | 704 | -4.256 | 0.0003 | 2.8039 | 2.6298 | 0.1532 | F (1, 120) = 22.5704, pFDR = 0.0000096385996, η² = 0.1542 | 0.1542 | 0.060325 | 0.734435 | 0.728094 |
|  | Right | transversetemporal | 50.5 | -15.5 | 4.5 | 224.39 | 494 | -4.294 | 0.002 | 2.5647 | 2.4253 | 0.1215 | F (1, 120) = 13.5422, pFDR = 0.0004111483, η² = 0.0993 | 0.0993 | 0.224598 | 0.892377 | 0.245613 |
|  | Right | supramarginal | 46.2 | -37.4 | 41.9 | 212.85 | 541 | -4.548 | 0.0031 | 2.4736 | 2.3262 | 0.1272 | F (1, 120) = 23.2054, pFDR = 0.000007688836, η² = 0.157 | 0.157 | 0.034403 | 0.973152 | 0.796906 |
|  | Right | superiortemporal | 45.8 | -34.5 | 12 | 191.87 | 437 | -4.917 | 0.0065 | 2.3093 | 2.1551 | 0.14 | F (1, 120) = 18.7114, pFDR = 0.000041367406, η² = 0.1327 | 0.1327 | 0.390753 | 0.958527 | 0.217727 |
|  | Right | superiorfrontal | 10.3 | 55.4 | 8.1 | 153.98 | 183 | -4.486 | 0.0203 | 2.8088 | 2.5752 | 0.2048 | F (1, 120) = 10.8787, pFDR = 0.0013604546, η² = 0.0775 | 0.0775 | 0.020326 | 0.26641 | 0.107275 |
|  | Right | parstriangularis | 51.5 | 31 | -1.4 | 146.68 | 271 | -4.556 | 0.0242 | 2.8253 | 2.6447 | 0.1663 | F (1, 120) = 19.2528, pFDR = 0.000033678639, η² = 0.1368 | 0.1368 | 0.252018 | 0.891731 | 0.761084 |
|  | Right | lateralorbitofrontal | 15.7 | 34.8 | -23.9 | 145.84 | 256 | -4.869 | 0.0248 | 2.9938 | 2.8215 | 0.152 | F (1, 120) = 12.9379, pFDR = 0.00053120275, η² = 0.0953 | 0.0953 | 0.137599 | 0.543295 | 0.68697 |
|  | Right | precentral | 55.4 | 3.2 | 32.1 | 122.31 | 258 | -4.242 | 0.0475 | 2.92 | 2.7446 | 0.167 | F (1, 120) = 14.0577, pFDR = 0.00033300291, η² = 0.1022 | 0.1022 | 0.206093 | 0.273042 | 0.398232 |
| **Volume** | |  |  |  |  |  |  |  |  |  |  |  |  |  |  |  |  |
|  | Left | superiorfrontal | -11.4 | 64 | -1.1 | 747.33 | 1259 | -5.317 | 0.0001 | 2.8512 | 2.5347 | 0.2239 | F (1, 120) = 23.7521, pFDR = 0.000023171927, η² = 0.1157 | 0.1157 | 3.31E-06 | 0.011693 | 1.44E-07 |
|  | Left | lateralorbitofrontal | -38.9 | 25.4 | -13.3 | 527.4 | 1049 | -5.280 | 0.0001 | 2.3415 | 2.0145 | 0.2299 | F (1, 120) = 23.1028, pFDR = 0.000023171927, η² = 0.1096 | 0.1096 | 0.000459 | 0.463892 | 2.47E-11 |
|  | Left | parstriangularis | -31.6 | 30 | 5.7 | 438.22 | 1068 | -5.496 | 0.0001 | 1.3189 | 1.1258 | 0.1393 | F (1, 120) = 15.2942, pFDR = 0.00021408309, η² = 0.0979 | 0.0979 | 0.000714 | 0.776831 | 0.003758 |
|  | Left | lateralorbitofrontal | -28.2 | 51.3 | -10.7 | 415.34 | 504 | -4.754 | 0.0001 | 3.5768 | 3.1895 | 0.3322 | F (1, 120) = 21.6984, pFDR = 0.000023171927, η² = 0.1214 | 0.1214 | 0.005921 | 2.78E-05 | 0.001861 |
|  | Left | middletemporal | -62 | -18.3 | -15.8 | 324.6 | 521 | -6.083 | 0.001 | 3.2096 | 2.6298 | 0.4422 | F (1, 120) = 21.7322, pFDR = 0.000023171927, η² = 0.1254 | 0.1254 | 0.001798 | 0.174002 | 2.22E-05 |
|  | Left | parsopercularis | -50.5 | 9.3 | 1.4 | 243.17 | 513 | -4.272 | 0.0049 | 1.9402 | 1.6909 | 0.2014 | F (1, 120) = 12.5975, pFDR = 0.00059493903, η² = 0.0855 | 0.0855 | 0.183752 | 0.70832 | 0.000493 |
|  | Left | superiorfrontal | -12.6 | 46.3 | 13.3 | 166.81 | 249 | -3.956 | 0.0334 | 2.6746 | 2.0844 | 0.4875 | F (1, 120) = 13.1142, pFDR = 0.00050234521, η² = 0.0914 | 0.0914 | 0.033582 | 0.133813 | 0.066846 |
|  | Right | lateralorbitofrontal | 41.1 | 25.4 | -13.3 | 645.6 | 1229 | -5.022 | 0.0001 | 2.1905 | 1.9215 | 0.1861 | F (1, 120) = 19.8206, pFDR = 0.000038362407, η² = 0.0958 | 0.0958 | 0.00017 | 0.128696 | 1.22E-10 |
|  | Right | superiorfrontal | 10.2 | 57.7 | 9 | 626.71 | 811 | -6.149 | 0.0001 | 3.2746 | 2.8724 | 0.2966 | F (1, 120) = 22.5138, pFDR = 0.000023171927, η² = 0.122 | 0.122 | 0.00141 | 0.432223 | 1.85E-07 |
|  | Right | lingual | 24.4 | -61 | -6.3 | 312.35 | 351 | -4.181 | 0.0007 | 2.5552 | 2.0397 | 0.6066 | F (1, 120) = 21.2975, pFDR = 0.000023171927, η² = 0.1453 | 0.1453 | 0.072527 | 0.225037 | 0.464465 |
|  | Right | middletemporal | 57.4 | -29.5 | -11.2 | 272.62 | 529 | -4.394 | 0.0027 | 1.6477 | 1.3875 | 0.1885 | F (1, 120) = 17.6846, pFDR = 0.000088384758, η² = 0.0979 | 0.0979 | 0.015974 | 0.850587 | 1.49E-08 |
|  | Right | postcentral | 62.2 | -11.7 | 25.6 | 270.95 | 693 | -4.223 | 0.0027 | 1.3311 | 1.1461 | 0.1351 | F (1, 120) = 13.3668, pFDR = 0.00048543443, η² = 0.0828 | 0.0828 | 0.011889 | 0.527454 | 1.09E-05 |
|  | Right | superiorfrontal | 10.1 | 41.2 | 46.8 | 221.38 | 421 | -4.492 | 0.0105 | 2.4166 | 2.1122 | 0.1951 | F (1, 120) = 11.4455, pFDR = 0.00096844934, η² = 0.0597 | 0.0597 | 0.00396 | 0.884881 | 6.46E-11 |
|  | Right | rostralmiddlefrontal | 23.9 | 45.1 | 19.9 | 180.8 | 301 | -4.510 | 0.0244 | 2.2198 | 1.7129 | 0.4479 | F (1, 120) = 16.9208, pFDR = 0.00011160549, η² = 0.1143 | 0.1143 | 0.082157 | 0.033726 | 0.068399 |

Table S 2. Connectome Results – detailed description

| Region A | Region B | Network A | Network B | Hemisphere A | Hemisphere B | Connection type | Effect Direction | Statistics (t) | p (FDR) |
| --- | --- | --- | --- | --- | --- | --- | --- | --- | --- |
| 17Networks_LH_SomMotA_4 | 17Networks_LH_DefaultB_PFCd_1 | SomMotA | DefaultB | L | L | Inter-network | HC > CUD | -4.653 | 4.56E-02 |
| 17Networks_RH_SomMotA_4 | 17Networks_RH_SalVentAttnA_FrMed_1 | SomMotA | SalVentAttnA | R | R | Inter-network | CUD > HC | 4.71 | 4.56E-02 |
| 17Networks_RH_SomMotA_6 | 17Networks_RH_SalVentAttnA_FrMed_1 | SomMotA | SalVentAttnA | R | R | Inter-network | CUD > HC | 4.743 | 4.56E-02 |
| 17Networks_RH_SomMotA_6 | 17Networks_RH_SalVentAttnA_FrMed_2 | SomMotA | SalVentAttnA | R | R | Inter-network | CUD > HC | 4.649 | 4.56E-02 |

Table S 3. Sensitivity analyses of group differences to clinical covariates

| Modality | Hemisphere | Region | Baseline | + Alcohol | + Education | + Alcohol + Education | + CTQ |
| --- | --- | --- | --- | --- | --- | --- | --- |
| Local Gyrification Index | Left | Lateral Orbitofrontal | <0.001 | 0.024 | 0.198 | 0.568 | 0.005 |
|  | Right | Precentral | 0.023 | 0.288 | 0.333 | 0.777 | 0.055 |
| Cortical Thickness | Left | Inferior Temporal | <0.001 | <0.001 | <0.001 | <0.001 | <0.001 |
|  | Left | Lateral Orbitofrontal | <0.001 | <0.001 | <0.001 | <0.001 | <0.001 |
|  | Left | Caudal Middle Frontal | <0.001 | <0.001 | <0.001 | <0.001 | <0.001 |
|  | Left | Rostral Middle Frontal | <0.001 | <0.001 | <0.001 | <0.001 | <0.001 |
|  | Left | Rostral Middle Frontal | <0.001 | <0.001 | <0.001 | 0.001 | <0.001 |
|  | Left | Supramarginal | <0.001 | <0.001 | <0.001 | 0.002 | <0.001 |
|  | Left | Rostral Middle Frontal | <0.001 | <0.001 | 0.005 | 0.013 | <0.001 |
|  | Left | Rostral Middle Frontal | <0.001 | <0.001 | 0.002 | 0.003 | <0.001 |
|  | Left | Fusiform | <0.001 | <0.001 | 0.003 | 0.005 | <0.001 |
|  | Left | Superior Parietal | <0.001 | <0.001 | <0.001 | <0.001 | <0.001 |
|  | Left | Superior Frontal | 0.003 | 0.033 | 0.057 | 0.229 | 0.004 |
|  | Left | Postcentral | <0.001 | <0.001 | 0.004 | 0.007 | <0.001 |
|  | Left | Postcentral | <0.001 | <0.001 | <0.001 | <0.001 | <0.001 |
|  | Left | Medial Orbitofrontal | <0.001 | <0.001 | <0.001 | <0.001 | <0.001 |
|  | Left | Lateral Occipital | 0.002 | 0.040 | 0.019 | 0.087 | 0.019 |
|  | Left | Lateral Occipital | <0.001 | <0.001 | <0.001 | <0.001 | <0.001 |
|  | Left | Precentral | <0.001 | 0.024 | 0.198 | 0.568 | 0.005 |
|  | Right | Middle Temporal | <0.001 | <0.001 | <0.001 | <0.001 | <0.001 |
|  | Right | Inferior Parietal | <0.001 | <0.001 | <0.001 | <0.001 | <0.001 |
|  | Right | Fusiform | <0.001 | <0.001 | <0.001 | <0.001 | <0.001 |
|  | Right | Postcentral | <0.001 | <0.001 | 0.004 | 0.007 | <0.001 |
|  | Right | Inferior Parietal | <0.001 | <0.001 | <0.001 | <0.001 | <0.001 |
|  | Right | Lateral Orbitofrontal | <0.001 | <0.001 | 0.001 | 0.006 | <0.001 |
|  | Right | Pars Opercularis | <0.001 | <0.001 | <0.001 | <0.001 | <0.001 |
|  | Right | Lateral Occipital | <0.001 | 0.003 | 0.014 | 0.052 | 0.003 |
|  | Right | Superior Parietal | <0.001 | 0.001 | 0.004 | 0.006 | 0.001 |
|  | Right | Postcentral | <0.001 | <0.001 | 0.003 | 0.007 | <0.001 |
|  | Right | Transverse Temporal | <0.001 | <0.001 | 0.001 | 0.002 | 0.002 |
|  | Right | Supramarginal | <0.001 | <0.001 | <0.001 | 0.001 | <0.001 |
|  | Right | Superior Temporal | <0.001 | <0.001 | 0.009 | 0.024 | <0.001 |
|  | Right | Superior Frontal | 0.001 | 0.007 | 0.013 | 0.022 | 0.002 |
|  | Right | Pars Triangularis | <0.001 | <0.001 | 0.018 | 0.020 | <0.001 |
|  | Right | Lateral Orbitofrontal | <0.001 | 0.019 | 0.004 | 0.036 | 0.001 |
|  | Right | Precentral | <0.001 | 0.005 | 0.013 | 0.029 | 0.002 |
| Cortical Surface-Based Volume | Left | Superior Frontal | <0.001 | <0.001 | 0.005 | 0.007 | <0.001 |
|  | Left | Lateral Orbitofrontal | <0.001 | <0.001 | 0.009 | 0.048 | <0.001 |
|  | Left | Pars Triangularis | <0.001 | <0.001 | 0.009 | 0.006 | <0.001 |
|  | Left | Lateral Orbitofrontal | <0.001 | <0.001 | 0.007 | 0.016 | <0.001 |
|  | Left | Middle Temporal | <0.001 | <0.001 | 0.004 | 0.003 | <0.001 |
|  | Left | Pars Opercularis | <0.001 | <0.001 | 0.007 | 0.004 | <0.001 |
|  | Left | Superior Frontal | <0.001 | 0.001 | 0.006 | 0.016 | <0.001 |
|  | Right | Lateral Orbitofrontal | <0.001 | <0.001 | 0.030 | 0.110 | <0.001 |
|  | Right | Superior Frontal | <0.001 | <0.001 | <0.001 | 0.003 | <0.001 |
|  | Right | Lingual | <0.001 | <0.001 | <0.001 | 0.002 | <0.001 |
|  | Right | Middle Temporal | <0.001 | <0.001 | <0.001 | <0.001 | <0.001 |
|  | Right | Postcentral | <0.001 | <0.001 | 0.043 | 0.036 | 0.001 |
|  | Right | Superior Frontal | 0.001 | 0.005 | 0.032 | 0.045 | 0.001 |
|  | Right | Rostral Middle Frontal | <0.001 | <0.001 | 0.006 | 0.008 | <0.001 |
|  |  |  |  |  |  |  |  |
| Edge | Connection (Schaefer-200) | | Baseline | + Alcohol (yrs) | + Education | + Alc + Edu | + CTQ |
| Functional Connectome | | | | | | | |
| 1 | LH SomMotA 4 <-> LH DefaultB PFCd 1 | | <0.001 | <0.001 | <0.001 | <0.001 | <0.001 |
| 2 | RH SomMotA 4 <-> RH SalVentAttnA FrMed 1 | | <0.001 | <0.001 | <0.001 | <0.001 | <0.001 |
| 3 | RH SomMotA 6 <-> RH SalVentAttnA FrMed 1 | | <0.001 | <0.001 | 0.002 | 0.002 | <0.001 |
| 4 | RH SomMotA 6 <-> RH SalVentAttnA FrMed 2 | | <0.001 | <0.001 | <0.001 | 0.002 | <0.001 |

Note. Each cell: p-value for the group effect (CUD vs HC), from a model with age, sex, eTIV (morphometry) or mean framewise displacement (connectome), plus the listed covariate added. Morphometry: FDR-BH–corrected within each model across the significant clusters. Connectome: uncorrected (4 edges). Baseline = published model with no added clinical covariate; group effects are reductions in CUD (morphometry) and the directions reported in the main results table (connectome).

Table S 4. Group × moderator interaction analyses

| Modality | Hemisphere | Region | Group × Sex | Group × Education | Group × Income | Group × CTQ |
| --- | --- | --- | --- | --- | --- | --- |
| Local Gyrification Index | Left | Lateral Orbitofrontal | 0.284 | 0.981 | 0.992 | 0.990 |
|  | Right | Precentral | 0.992 | 0.823 | 0.650 | 0.990 |
| Cortical Thickness | Left | Inferior Temporal | 0.992 | 0.823 | 0.450 | 0.990 |
|  | Left | Lateral Orbitofrontal | 0.992 | 0.859 | 0.992 | 0.990 |
|  | Left | Caudal Middle Frontal | 0.992 | 0.859 | 0.993 | 0.990 |
|  | Left | Rostral Middle Frontal | 0.992 | 0.773 | 0.929 | 0.990 |
|  | Left | Rostral Middle Frontal | 0.992 | 0.823 | 0.450 | 0.990 |
|  | Left | Supramarginal | 0.284 | 0.981 | 0.450 | 0.990 |
|  | Left | Rostral Middle Frontal | 0.992 | 0.859 | 0.450 | 0.990 |
|  | Left | Rostral Middle Frontal | 0.992 | 0.859 | 0.992 | 0.990 |
|  | Left | Fusiform | 0.689 | 0.859 | 0.929 | 0.990 |
|  | Left | Superior Parietal | 0.992 | 0.966 | 0.450 | 0.990 |
|  | Left | Superior Frontal | 0.992 | 0.902 | 0.185 | 0.990 |
|  | Left | Postcentral | 0.992 | 0.859 | 0.992 | 0.990 |
|  | Left | Postcentral | 0.992 | 0.823 | 0.929 | 0.990 |
|  | Left | Medial Orbitofrontal | 0.284 | 0.859 | 0.929 | 0.990 |
|  | Left | Lateral Occipital | 0.992 | 0.953 | 0.993 | 0.990 |
|  | Left | Lateral Occipital | 0.992 | 0.981 | 0.999 | 0.990 |
|  | Left | Precentral | 0.284 | 0.981 | 0.992 | 0.990 |
|  | Right | Middle Temporal | 0.992 | 0.953 | 0.185 | 0.990 |
|  | Right | Inferior Parietal | 0.992 | 0.818 | 0.929 | 0.990 |
|  | Right | Fusiform | 0.992 | 0.981 | 0.687 | 0.990 |
|  | Right | Postcentral | 0.992 | 0.859 | 0.992 | 0.990 |
|  | Right | Inferior Parietal | 0.992 | 0.859 | 0.302 | 0.990 |
|  | Right | Lateral Orbitofrontal | 0.992 | 0.739 | 0.687 | 0.990 |
|  | Right | Pars Opercularis | 0.992 | 0.981 | 0.992 | 0.990 |
|  | Right | Lateral Occipital | 0.284 | 0.773 | 0.450 | 0.990 |
|  | Right | Superior Parietal | 0.992 | 0.890 | 0.992 | 0.990 |
|  | Right | Postcentral | 0.992 | 0.859 | 0.687 | 0.990 |
|  | Right | Transverse Temporal | 0.992 | 0.859 | 0.929 | 0.990 |
|  | Right | Supramarginal | 0.992 | 0.981 | 0.450 | 0.990 |
|  | Right | Superior Temporal | 0.992 | 0.818 | 0.992 | 0.990 |
|  | Right | Superior Frontal | 0.992 | 0.981 | 0.450 | 0.990 |
|  | Right | Pars Triangularis | 0.992 | 0.818 | 0.929 | 0.990 |
|  | Right | Lateral Orbitofrontal | 0.992 | 0.890 | 0.929 | 0.990 |
|  | Right | Precentral | 0.423 | 0.859 | 0.929 | 0.990 |
| Cortical Surface-Based Volume | Left | Superior Frontal | 0.992 | 0.981 | 0.992 | 0.990 |
|  | Left | Lateral Orbitofrontal | 0.992 | 0.981 | 0.992 | 0.990 |
|  | Left | Pars Triangularis | 0.992 | 0.739 | 0.450 | 0.990 |
|  | Left | Lateral Orbitofrontal | 0.992 | 0.902 | 0.929 | 0.990 |
|  | Left | Middle Temporal | 0.992 | 0.871 | 0.929 | 0.990 |
|  | Left | Pars Opercularis | 0.992 | 0.859 | 0.992 | 0.990 |
|  | Left | Superior Frontal | 0.992 | 0.823 | 0.929 | 0.990 |
|  | Right | Lateral Orbitofrontal | 0.992 | 0.773 | 0.711 | 0.990 |
|  | Right | Superior Frontal | 0.992 | 0.890 | 0.929 | 0.990 |
|  | Right | Lingual | 0.992 | 0.966 | 0.324 | 0.990 |
|  | Right | Middle Temporal | 0.277 | 0.739 | 0.984 | 0.440 |
|  | Right | Postcentral | 0.992 | 0.859 | 0.929 | 0.990 |
|  | Right | Superior Frontal | 0.992 | 0.739 | 0.992 | 0.990 |
|  | Right | Rostral Middle Frontal | 0.992 | 0.852 | 0.534 | 0.990 |
|  |  |  |  |  |  |  |
| Functional Connectome | | | | | | |
| Edge | Connection (Schaefer-200) | | Group × Sex | Group × Education | Group × Income | Group × CTQ |
| 1 | LH SomMotA 4 - LH DefaultB PFCd 1 | | 0.169 | 0.091 | 0.199 | 0.387 |
| 2 | RH SomMotA 4 - RH SalVentAttnA FrMed 1 | | 0.591 | 0.692 | 0.111 | 0.540 |
| 3 | RH SomMotA 6 - RH SalVentAttnA FrMed 1 | | 0.219 | 0.246 | 0.291 | 0.649 |
| 4 | RH SomMotA 6 - RH SalVentAttnA FrMed 2 | | 0.485 | 0.926 | 0.397 | 0.451 |

Note. Each cell: p-value for the group × moderator interaction. Moderators: sex; education and income (socioeconomic status); CTQ total (early-life stress). Continuous moderators mean-centered; models adjusted for age, sex, eTIV (morphometry) or mean framewise displacement (connectome). Morphometry: FDR-BH–corrected within each moderator across the significant clusters. Connectome: uncorrected (4 edges). No interaction survived FDR correction.

Table S 5. Signal-to-noise ratio (SNR) of hippocampal and amygdala subfields

| **Subfield** | **Structure** | **CUD SNR (mean ± SD)** | **HC SNR (mean ± SD)** | **t (df)** | **p** | **SNR rank** |
| --- | --- | --- | --- | --- | --- | --- |
| Molecular layer (body) | Hippocampus | 16.24 ± 1.77 | 17.17 ± 1.39 | -3.31 (123) | 0.001 | 13/28 |
| CA1 (head) | Hippocampus | 15.59 ± 1.51 | 16.33 ± 0.99 | -3.31 (121) | 0.001 | 8/28 |
| GC-ML-DG (head) | Hippocampus | 16.71 ± 1.78 | 17.68 ± 1.52 | -3.31 (120) | 0.001 | 17/28 |
| Molecular layer (head) | Hippocampus | 16.45 ± 1.84 | 17.38 ± 1.49 | -3.11 (122) | 0.002 | 15/28 |
| CA4 (head) | Hippocampus | 19.31 ± 2.38 | 20.52 ± 2.15 | -2.97 (118) | 0.004 | 25/28 |
| Corticoamygdaloid transition | Amygdala | 14.02 ± 1.41 | 14.77 ± 1.38 | -2.96 (114) | 0.004 | 6/28 |
| Hippocampal-amygdaloid transition area (HATA) | Hippocampus | 13.16 ± 1.59 | 13.89 ± 1.38 | -2.75 (120) | 0.007 | 1/28 |
| Subiculum (head) | Hippocampus | 16.22 ± 1.83 | 17.06 ± 1.67 | -2.66 (117) | 0.009 | 12/28 |
| CA3 (head) | Hippocampus | 13.95 ± 1.90 | 14.49 ± 1.71 | -1.66 (118) | 0.099 | 4/28 |
| Presubiculum (head) | Hippocampus | 18.59 ± 2.19 | 18.85 ± 1.85 | -0.70 (121) | 0.483 | 21/28 |

Note. SNR was computed as the mean/standard-deviation of the normalized T1-weighted intensity within each FreeSurfer subfield label, averaged across hemispheres (CUD n = 72; HC n = 53). Groups were compared with Welch's two-sample t-test. SNR rank orders all 28 segmented subfields from lowest (1) to highest SNR. SNR was lower in the CUD group in 10 of 10 subfields and significantly lower (p < 0.05) in 8 of 10; the hippocampal-amygdaloid transition area (HATA) showed the lowest SNR overall. These subfields are the regions in which exploratory volumetric differences were observed; their intrinsically low and group-differential SNR indicates the subfield findings should be regarded as exploratory.

Table S 6. Structure–function convergence analyses using within-individual coupling

| Structural score | β (structure) | p | Structure × group p | Within-CUD r (p) | Within-HC r (p) |
| --- | --- | --- | --- | --- | --- |
| Overall | +0.015 | 0.955 | 0.904 | +0.05 (0.678) | +0.03 (0.828) |
| Thickness | +0.042 | 0.866 | 0.813 | +0.09 (0.481) | +0.07 (0.620) |
| Volume | -0.084 | 0.688 | 0.841 | -0.04 (0.776) | -0.08 (0.588) |
| LGI | +0.017 | 0.887 | 0.478 | +0.04 (0.753) | +0.15 (0.302) |

Note. Cross-validated PLS (structural clusters × whole-connectome node degree, 5-fold cross-validation, non–group-selected): mean |latent r| = 0.20, permutation p = 0.27.

Table S 7. Structure–function convergence analyses using targeted cross-modal partial least squares

| Targeted Region | Loading |
| --- | --- |
| Function | |
| e1 - L somatomotor ↔ L dorsolateral-PFC (hypo) | -0.424 |
| e2 - R somatomotor ↔ R medial-frontal (hyper) | +0.456 |
| e3 - R somatomotor ↔ R medial-frontal (hyper) | +0.584 |
| e4 - R somatomotor ↔ R medial-frontal (hyper) | +0.521 |
| Structure | |
| Left Supramarginal (Thickness) | −0.266 |
| Right Inferior Parietal (Thickness) | −0.243 |
| Left Medial Orbitofrontal (Thickness) | −0.237 |
| Left Pars Opercularis (Volume) | −0.233 |
| Right Pars Triangularis (Thickness) | −0.231 |
| Left Superior Frontal (Volume) | −0.229 |
| Right Inferior Parietal (Thickness) | −0.219 |
| Right Middle Temporal (Volume) | −0.185 |
| Left Rostral Middle Frontal (Thickness) | −0.180 |
| Right Superior Temporal (Thickness) | −0.170 |

Note. Because both blocks (clusters, edges) were selected on the group contrast, this analysis is partially circular and describes the joint structural-functional pattern rather than providing independent evidence of coupling. Blocks were residualized for age, sex, and mean framewise displacement and z-scored feature-wise before decomposition. First latent variable (LV1): 92.1% of the cross-covariance between the structural and functional blocks; latent correlation r = 0.42; permutation p < 0.001 (2,000 permutations).

Figure S 1. Density scatter of the functional group-effect magnitude (|t|, CUD vs. HC) across all 19,900 edges against each edge's proximity to structurally altered cortex (distance from the Schaefer-200 node centroid to the nearest structural-cluster peak). The two maps were weakly associated (Spearman ρ = 0.05), significant under a node-permutation null (p = 0.007) but not under a spatially constrained spin null (p = 0.09). Because non-spin spatial permutations are inflated by spatial autocorrelation, the spatial correspondence between the structural and functional phenotypes should be regarded as suggestive at most.
